# PLA-Based Biodegradable Polymer from Synthesis to the Application

**DOI:** 10.3390/polym18010121

**Published:** 2025-12-31

**Authors:** Junui Wi, Jimin Choi, Sang-Ho Lee

**Affiliations:** Department of Chemical and Biochemical Engineering, Dongguk University, Seoul 04620, Republic of Korea

**Keywords:** Poly(lactic acid), synthesis mechanism, enhancing properties, stereocomplex, blending, copolymerization, application

## Abstract

Poly(lactic acid) (PLA) has emerged as a leading bio-based polymer due to its renewability, processability, and biodegradability, yet its broader adoption remains constrained by limitations in thermal stability, mechanical performance, and end-of-life control. This review provides a comparative and application-oriented overview of recent advances in PLA from synthesis and catalyst landscapes to structure–property–biodegradation relationships and practical applications. Representative polymerization routes and catalyst systems are critically compared in terms of achievable molecular weight, stereochemical control, scalability, and sustainability. Key structure–property modification strategies—including stereocomplex formation, blending, and copolymerization—are quantitatively evaluated with respect to thermal and mechanical properties, highlighting inherent trade-offs. Importantly, environment-specific biodegradation behaviors are assessed using representative quantitative metrics under industrial composting, soil, marine, and enzymatic conditions, underscoring the strong dependence of degradation on both material design and testing environment. Finally, application-driven requirements for food packaging, fibers, and agricultural materials are discussed alongside regulatory considerations, processing constraints, and qualitative cost positioning relative to conventional polymers. By integrating recent representative studies into comparative tables and synthesis-driven discussions, this review offers design guidelines for tailoring PLA-based materials toward targeted performance and sustainable deployment.

## 1. Introduction

Global plastic production has surged in recent decades, totaling over 8300 million tons even been produced to date. However, less than 9% is recycled, with most ending up in landfills, incinerators, or accumulating in the environment, causing serious ecological problems [1]. Plastic entering the natural environment degrades into microplastics, causing significant concern as it disrupts ecosystems and can enter the human body through the food chain [2]. These global waste and carbon emission problems demand a comprehensive reevaluation of the existing plastic lifecycle system. Consequently, interest in biodegradable and bio-based polymers is rapidly increasing [3].

Poly(lactic acid) (PLA) has established itself as one of the most extensively researched and commercially successful bio-based biodegradable polymers. PLA is a fully bio-based polymer derived from lactic acid, obtained by fermenting plant-derived carbohydrates such as starch and cellulose. Leveraging its high transparency, excellent mechanical strength, biocompatibility, and biodegradability, it is utilized across diverse fields, including packaging materials, films, fibers, and medical and bio-materials [4,5]. PLA is particularly regarded as a key material in medical applications such as tissue engineering scaffolds, sutures, and drug delivery systems because it degrades into lactic acid in the body [6].

However, PLA inherently possesses structural limitations such as brittleness, low heat deflection temperature, moderate gas barrier properties, and relatively slow biodegradation rates, which constrain its application as a high-performance industrial material. Therefore, strategies such as stereocomplex (SC) formation, copolymerization, and blending are being employed [7,8]. Furthermore, various catalysts—ranging from Al, Mg, and Zn-based catalysts to metal-free organocatalysts and Lewis pair systems—are known to decisively influence PLA polymerization characteristics (stereoregularity, molecular weight, and molecular weight distribution [MWD]) [9].

PLA is primarily manufactured via direct polycondensation and lactide-based ring-opening polymerization (ROP), with ROP becoming the industrial standard for its high molecular weight, narrow MWD, and controllable stereoregularity [10]. In ROP, the acid–base properties, electronic structure, and steric factors of the catalyst significantly influence the active site’s reactivity. Metal-free catalysts, in particular, are actively researched in academia as they avoid residual metal toxicity issues. The PLA synthesis route and catalyst system directly determine the microstructure and properties of the final polymer, making polymerization design and catalyst control essential for enhancing PLA functionality. Furthermore, PLA applications are rapidly expanding beyond traditional packaging materials, fibers, films, and agricultural supplies into high-performance industrial materials, 3D printing, smart textiles, and medical biomaterials [11].

This review aims to synthesize existing research on PLA from an integrated perspective encompassing Synthesis–Structure–Property–Application, thereby providing a comprehensive understanding of PLA-based biodegradable polymers throughout their entire lifecycle. Specifically, by organically linking lactic acid production and lactide manufacturing, polymerization pathways and catalyst systems, property modification strategies, and industrial applications, this review aims to comprehensively survey the current technological level of PLA and propose future technical challenges and research directions. Furthermore, it provides a balanced overview of PLA’s advantages and limitations, laying the groundwork for simultaneously addressing environmental issues and developing high-value-added bio-based polymers.

## 2. PLA Synthesis

PLA is a polymer obtained from lactic acid monomers, broadly categorized into direct polycondensation and ROP via lactide, depending on the monomer polymerization method. Direct polycondensation is a pathway where lactic acid is directly esterified to form PLA, subdivided into melt (bulk) polycondensation, solution polycondensation, and melt-solid polycondensation. In this pathway, efficient removal of water generated during the reaction is essential to achieve high molecular weight and yield. Nevertheless, it inherently faces limitations in achieving molecular weights (<50,000 g/mol) and securing a narrow MWD.

In contrast, ROP using lactide as an intermediate, despite its relatively complex process and increased cost, has established itself as the mainstream route for industrial PLA synthesis due to its distinct advantages in high molecular weight (≥50,000 g/mol), narrow MWD, and tacticity control. In lactide ROP, metal catalysts or metal-free catalysts (organic catalysts, enzyme catalysts, Lewis acids/bases, etc.) are primarily used. Recently, research on metal-free catalysts and non-tin metal catalysts has been actively progressing due to toxicity and regulatory concerns regarding metal residues. This chapter summarizes the direct lactic acid condensation polymerization route and its modified processes. Additionally, it provides an overview of key strategies for PLA synthesis, focusing on catalyst types, mechanisms, and recent trends in ROP.

### 2.1. Polycondensation

**Figure 1:**
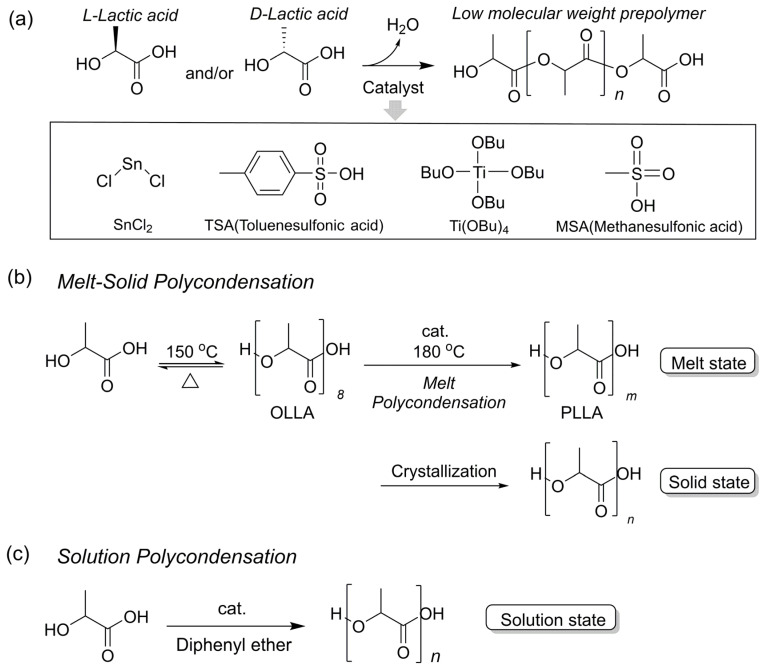
(**a**) General synthetic mechanism of PLA from lactic acid using different catalysts, (**b**) melt-solid polycondensation, and (**c**) solution poly condensation.

Direct condensation polymerization of lactic acid is classified as a step-growth reaction. The hydroxyl and carboxyl groups of two lactic acid molecules undergo a condensation reaction to form an ester bond, with water generated as a byproduct during this process (Figure 1a). The generated water induces a side reaction: hydrolysis recuts the already formed ester bond, reverting it to monomer or oligomer. Therefore, continuous removal of moisture from the reaction system is essential for synthesizing high molecular weight PLA.

Various process modifications have been proposed for direct polycondensation, such as combining vacuum conditions, moisture removal using high-temperature environments, or post-reaction treatment processes. Direct polycondensation is categorized into melt (bulk) polycondensation, melt-solid polycondensation, and solution polycondensation, depending on the process conditions, catalyst used, and reaction medium. Each process exhibits distinct advantages and disadvantages in terms of processability, economics, molecular weight, and stereoregularity. Meanwhile, since lactic acid possesses L/D enantiomers, the stereochemical composition of the monomers used in direct polycondensation can result in PLA with stereostructures such as poly(L-lactide) (PLLA), poly(D-lactide) (PDLA), or poly(*rac*-lactide) PDLLA.

The initial research on the direct condensation polymerization of L-lactic acid was initiated by the Mitsui group. The initial process by Suzuki et al. [12] involves a solution polycondensation method using high-boiling-point organic solvents (e.g., diphenyl ether) in an azeotropic inert environment with SnO or SnCl_2_ catalysts. This process has limitations, including high solvent costs, difficulties in solvent–product separation, and reduced economic viability due to long reaction times.

Therefore, the Kimura group introduced a solvent-free melt (bulk) polycondensation process [13]. In this process, lactic acid is first dehydrated at high temperatures to form low-molecular-weight oligo(L-lactic acid). Subsequently, direct condensation is performed in the molten state under various catalysts to obtain high-molecular-weight PLA (Figure 1b). They demonstrated that high molecular weight PLA can be obtained in a relatively short time using only melt polycondensation, reporting that conditions using a binary catalyst of Sn(II) catalyst and strong acid (p-toluenesulfonic acid [TSA]) are the most efficient. When using SnCl_2_ alone, side reactions such as racemization, yellowing, and lactide formation increase; therefore, it is important to use a cocatalyst such as TSA or PPh_3_ in combination [14]. Additionally, the Kimura group combined SnCl_2_ with various metal alkoxides and proposed that Ge(OEt)_4_ is the most efficient cocatalyst [15]. Titanium alkoxide, particularly Ti(OBu)_4_, has also been reported as an effective single-based catalyst in melt polycondensation [16]. However, while Ti(OR)_4_-based catalysts exhibit high catalytic activity, they have the limitation of actively inducing ester exchange (alkoxy exchange), which can disrupt the microstructure [17].

Melt-solid polycondensation is a three-step process combining melt polycondensation and solid-state polymerization (SSP) (Figure 1b). First, short-term melt polycondensation is conducted around 180 °C to obtain low-molecular-weight PLLA. This is then crystallized at 105 °C, followed by subsequent polycondensation in the solid state within the temperature range above the glass transition temperature (*T*_g_) and below the melting point (*T*_m_). This process, proposed by the Kimura group to address the high-cost solvent use and long reaction times associated with Suzuki et al.‘s solution polycondensation, gained attention as an industrial alternative for obtaining high-molecular-weight PLA without solvent use [18]. Kimura group reported that a 1:1 combination of SnCl·2H_2_O and PPh·TSA is the most efficient catalyst combination in the melt phase. They demonstrated that SSP can achieve very high molecular weights of *M*_w_ ≈ 600,000 without racemization. This process offers the advantage of significantly reducing yellowing—a problem in melt polycondensation—while greatly enhancing molecular weight.

In the 1980s, the Suzuki Group developed solution polycondensation using high-boiling-point organic solvents such as diphenyl ether and toluene, aiming for mass production of PLA. This process is an acid-catalyzed esterification-based reaction. It involves dissolving lactic acid in a high-boiling-point solvent such as diphenyl ether or toluene, then continuously removing water generated during the reaction using a Dean–Stark apparatus, while employing a strong acid (such as TSA or Methanesulfonic acid (MSA)) or a Lewis acid (such as SnCl_2_) as a catalyst (Figure 1c) [12]. However, the solution process suffers from reduced reaction rates due to monomer dilution in the solvent, and its competitiveness is low compared to melt-solid polycondensation and lactide ROP in terms of processability and economics due to the use of expensive solvents and the difficulties in solvent recovery and purification. Therefore, solution polycondensation is rarely used in industrial processes today and is primarily limited to laboratory-scale studies for process comparison and mechanism research.

In summary, direct condensation polymerization faces fundamental limitations: it requires continuous removal of the byproduct water and necessitates high-temperature, long-duration reactions. These constraints hinder the simultaneous achievement of high molecular weight and a narrow MWD. Lactide-based ring-opening polymerization is the main method for PLA synthesis due to its lack of byproducts and ability to achieve high molecular weights.

### 2.2. Ring-Opening Polymerization (ROP)

#### 2.2.1. ROP Using Metal Catalysts

**Figure 2:**
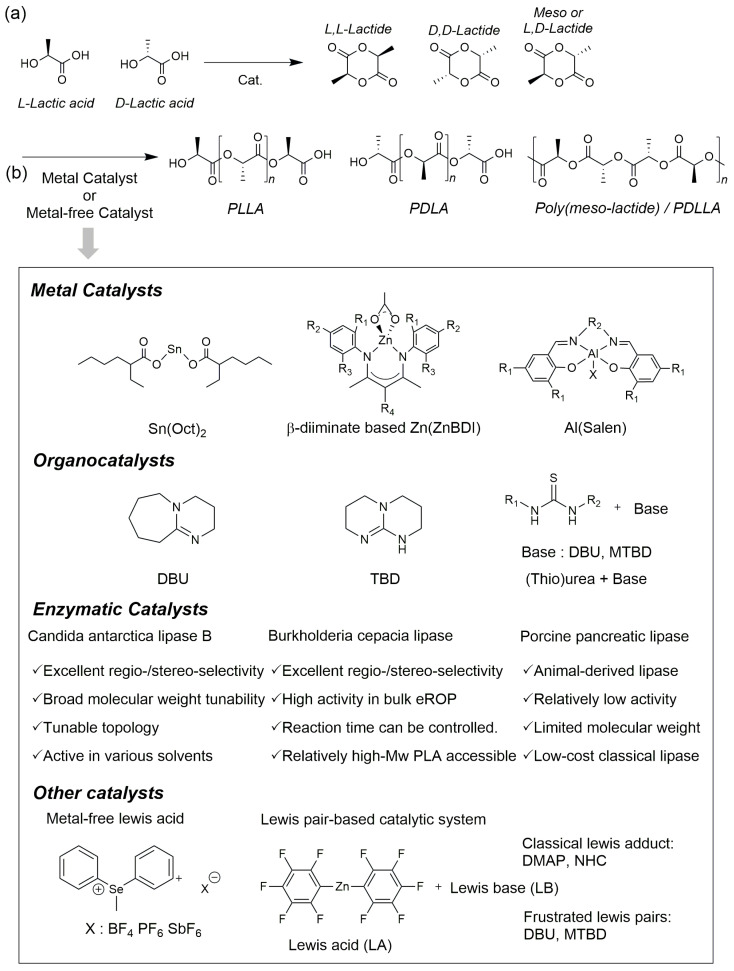
(**a**) General ring-opening polymerization mechanism of PLA from lactic acid, and (**b**) the metal and metal-free catalysts used.

In metal catalyst-based PLA ROP, it is generally understood that the metal-alkoxide active species coordinates the lactide monomer to the Lewis acidic metal center, followed by chain growth via a coordination-insertion mechanism involving acyl-oxygen bond cleavage (Figure 2) [9]. Many Sn-, Zn-, and Al-based catalysts employed for PLA polymerization operate through metal–alkoxide active species, where differences in metal identity govern the balance between catalytic robustness, stereochemical control, and sensitivity to processing conditions. Kohn et al. experimentally supported the coordination–insertion mechanism at the metal–alkoxide center through complex formation studies with metal precursors such as Sn(ΙΙ) 2-ethylhexanoate (Sn(Oct)_2_), AlBr_3_, and Triisobutylaluminum (TIBA), along with reaction rate and molecular weight analyses [19]. They explained that during the initiation step, the added alcohol (ROH) combines with the metal to form a metal–OR (M–OR) species. Subsequently, the lactide’s carbonyl group coordinates to the metal center, and the lactide ring undergoes cyclization through an electron rearrangement involving the M–OR bond [20,21]. During the reaction, alcohol or water acts as a chain transfer agent, enabling the “immortal ROP” characteristic where a single metal center equilibrates with multiple growing chains [22,23]. This metal catalyst-based ROP process is gaining interest in academia and industry for its advantages: high catalytic activity, high conversion rates, high molecular weights near the target, narrow MWD, and the capability for stereoselectivity based on conditions.

Latest commercial PLA production relies on bulk ROP of L-lactide (LLA), with process conditions typically set at high temperatures above 190 °C, under solvent-free conditions, and with an LA·catalyst molar ratio of approximately 10,000:1. Even under these conditions, achieving high conversion rates, high molecular weight (>100 kg/mol), narrow MWD, and low residual catalyst content simultaneously remains a key requirement for industrial catalyst design (Figure 2b). Accordingly, Sn(Oct)_2_ has long been used as the industrial standard. Concerns over residual tin toxicity have led to active development of catalysts using less toxic metals such as Zn and Al [24].

However, despite these toxicity concerns, Sn(Oct)_2_ remains the most widely used ROP catalyst industrially. Sn(Oct)_2_ is a precursor catalyst, and the actual active species is considered to be the Sn(II)-alkoxide formed by alcoholysis with alcohol [25]. Sn(II)–alkoxide follows a typical coordination–insertion pathway where lactide coordinates to the metal center, followed by insertion of the acyl–oxygen into the Sn–OR bond. The combination of Sn(Oct)_2_ and alcohols enables the implementation of immortal ROP under conditions containing numerous protic species, offering the advantage of achieving high productivity even at relatively low catalyst concentrations [26]. This pathway facilitates high conversion rates, fast reaction speeds, and the attainment of high molecular weights, enabling the stable production of PLA under industrial process conditions (180–200 °C).

However, when lactide is polymerized at high temperatures for extended periods with alcohol using a metal catalyst, it induces back-biting and various transesterification reactions, increasing the polydispersity index (PDI) [27]. Therefore, alcoholysis influences catalytic activity and molecular weight during ROP by acting as a chain-transfer process that terminates growing chains and regenerates metal–alkoxide species, thereby increasing chain number while limiting chain length. Furthermore, during transesterification, the stereochemical arrangement at the chain ends becomes random, leading to epimerization, where LLA units convert to LDA units, and a decrease in stereoregularity [28]. These effects directly impact PLA tacticity and SC formation. Therefore, precise optimization of temperature, reaction time, catalyst concentration, and co-initiator type (e.g., alcohol) is essential in designing Sn-catalyzed PLA ROP processes. The toxicity of tin species renders Sn catalysts unsuitable for medical-grade PLA synthesis, and the need for extra catalyst removal processes is a notable limitation.

Against this backdrop, non-tin metal catalysts are being actively researched as alternatives to replace Sn catalysts. Magnesium (Mg), an alkaline earth metal in Group 2, is gaining attention as a candidate catalyst for PLA ROP due to its low toxicity and excellent biocompatibility. Zinc (Zn) is also being extensively studied as an Sn replacement catalyst owing to its low toxicity, abundant availability, and similar coordination number and oxidation state to Mg. The Coates group demonstrated that Zn(II) complexes bearing *β*-diiminate (BDI) ligands exhibit high catalytic activity and excellent stereoselectivity in L-/D-/*rac*-lactide ROP, following a coordination–insertion mechanism similar to that of Sn(II) [29]. Various tacticity control strategies, including isoselective/heteroselective polymerization and stereoblock PLA synthesis, have been proposed through electronic structure tuning [30]. Furthermore, Zn complexes based on diverse N,O-donor ligands such as bisguanidine, pyridylamido, aminophenolate, and guanidinate have been developed [31,32]. Most of these are reported to exhibit a turnover frequency (100–10,000 h^−1^) capable of completely converting LLA within minutes under solution conditions at temperatures ranging from room temperature to 80 °C [31]. The Pellecchia group designed Zn(II) complexes with bis(iminopyridyl) or guanidinate ligands and compared their activity not only under solution conditions but also under melt conditions mimicking industrial conditions (190 °C, unrefined technical-grade monomer, very low catalyst concentration) [33]. The results showed that the catalyst exhibiting the highest activity under bulk conditions at room temperature exhibited reduced performance under high-temperature melt conditions. Conversely, a catalyst showing moderate activity under mild conditions provided high activity comparable to Sn(Oct)_2_ and yielded high molecular weight PLA [34]. Furthermore, Wood et al. demonstrated that the same Zn catalyst family could depolymerize commercial PLLA in the presence of alcohols to convert it into useful intermediates such as methyl lactate. This demonstrated the potential for Zn catalysts to serve as a useful platform for the chemical recycling of PLA [35,36]. However, most Zn catalysts exhibit limitations in activity reduction under high-temperature industrial conditions, and those suitable for large-scale production are still being developed [34]. More broadly, Zn-based catalysts, despite their low toxicity and earth abundance, often suffer from moderate Lewis acidity and sensitivity to coordinating impurities such as moisture and alcohols, which can complicate precise control over polymerization kinetics and stereochemistry, particularly under less strictly controlled conditions.

Aluminum(III)-based catalysts are one of the main groups actively studied in the lactone ROP field, based on advantages such as abundant resources in the Earth’s crust, low toxicity, and high Lewis acidity [9]. Spassky et al. first reported stereoselective ROP polymerization using binaphthyl Schiff base/Al alkoxide initiators [37]. Additionally, Coates demonstrated the high effectiveness of Al catalysts for stereoselective PLA polymerization by synthesizing stereoblock PLA using racemic Al-alkoxide [38]. Al catalysts also follow a coordination–insertion mechanism, similar to Sn and Zn catalysts, where they react with alcohols to form Al–OR active species. Lactide then coordinates to the Lewis acidic Al center, and polymerization proceeds via acyl-oxygen insertion into the Al–OR bond [39]. However, Al-based catalysts are generally sensitive to moisture. Exposure to air or moisture readily deactivates them via hydrolysis of the Al-OR bond. Some also suffer from decomposition at high temperatures or ligand departure. Consequently, direct application to large-scale industrial processes remains limited. This limitation can be partially mitigated through rigorous drying protocols, in situ alkoxide formation, or ligand design that enhances steric protection and hydrolytic stability.

#### 2.2.2. ROP Using Organic Catalysts

Metal-free catalysts encompass various series of catalysts, but among these, organic catalysts are the most actively researched field. Organic catalysts serve as an alternative to perform ROP, avoiding the residual metal issues associated with metal catalysts (Figure 2b). They offer advantages such as easy catalyst removal, excellent accessibility, superior chemical selectivity, high functional group tolerance, and the potential to control polymer structure [40]. Meanwhile, Zinck et al. reported that applying strong base organic catalysts to PLA ROP can induce side reactions such as transesterification, epimerization, and chain scission, potentially reducing PLA’s optical purity or affecting its thermal properties [41]. Therefore, various organic catalyst systems have been proposed, including N-heterocyclic carbene (NHC) [42], 4-(N,N-dimethylamino)pyridine (DMAP) [43], 1,8-diazabicyclo(5.4.0)undec-7-ene (DBU), 1,5,7-triazabicyclo(4.4.0)dec-5-ene (TBD) [44], and (thio)urea [45].

DBU is a strong base metal-free organic catalyst with a bicyclic amidine structure. It is advantageous for the deprotonation of alcohols and, based on its large steric hindrance and low nucleophilicity, tends to directly attack ester bonds less frequently compared to other organic catalysts. Thus, it suppresses unnecessary side reactions better than other strong base catalysts, making it one of the organic catalysts receiving significant research attention [46]. Hedrick et al. investigated TBD, 7-methyl-1,5,7-triazabicyclo(4.4.0)dec-5-ene (MTBD), and DBU, demonstrating excellent control over molecular weight and polydispersity in ROP of cyclic esters. These catalysts are suitable for lactide ROP due to their excellent solubility, mild polymerization conditions, and metal-free process [44]. Subsequently, the Hedrick group demonstrated that performing lactide ROP with DBU alone, without an alcohol initiator, can generate cyclized PLA via a zwitterionic mechanism. This suggested that DBU-catalyzed ROP involves not only the alcohol activation pathway but also a zwitterionic ring-opening pathway initiated by direct nucleophilic attack from DBU [47]. Sherck and Won integrated these mechanisms, proposing a unified mechanism and kinetic model for DBU-catalyzed lactide ROP. They suggested that the activated-alcohol pathway, where polymerization occurs via alcohol activation, and the nucleophilic-attack pathway, where polymerization occurs via direct nucleophilic attack of DBU on the lactide monomer, coexist competitively [48]. Recently, studies have reported utilizing DBU as a precision ROP platform to design and synthesize PLA with complex topologies such as linear, star, and comb structures [49]. This functionality is achieved using polar solvents, low-temperature conditions, and a starve-fed method involving repeated monomer addition. Therefore, DBU can be used as a multifunctional organic catalyst platform that enables both reaction pathway design and control of polymer microstructure/topology, extending beyond a simple strong base catalyst.

TBD is a strong base metal-free organic catalyst with a bicyclic guanidine skeleton [50]. It follows a dual activation mechanism: N–H hydrogen bonding activates the lactide carbonyl group while the basic nitrogen deprotonates the alcohol initiator [51]. This enables favorable short reaction times, predictable molecular weights, and narrow MWD even at lower catalyst concentrations and under mild conditions, representing a unique advantage of TBD compared to DBU [44]. According to the Hedrick group’s report, TBD functions as a highly active catalyst providing high conversion and narrow distribution even under mild conditions near room temperature in ROP of lactide and various cyclic esters. This prompted the full-scale expansion of research on guanidine-based organic catalysts [44]. Rice et al. analyzed LLA ROP using guanidine-based catalysts through experiments. They established the mechanistic basis for TBD by demonstrating that a dual activation mechanism via hydrogen bonding is thermodynamically and kinetically more favorable than a mechanism based on acetyl transfer to the catalyst [51]. Subsequent studies have emphasized TBD’s capability for stereoselective polymerization. The Coulembier group demonstrated that achiral TBD alone can yield isotactic or heterotactic PLA from rac- and meso-lactide ROP, depending on reaction temperature and monomer type. They also highlighted that stereoselective polymerization is achievable via chain-end control, even with a non-chiral catalyst [52]. This research trajectory shows that TBD has transformed from a highly active metal-free ROP catalyst into a versatile organic catalyst platform, enabling stereoselective polymerization through dual activation and precise adjustment of PLA microstructure.

Unlike DBU or TBD, (thio)urea-based catalysts act as hydrogen bond donors rather than strong bases/nucleophiles. Consequently, while their reaction rates are slower, they rarely induce side reactions such as racemization, basic decomposition, or transesterification [53]. Therefore, in situations where precise structural control and living ROP characteristics are more critical than speed, (thio)urea-based hydrogen bond catalyst systems are more advantageous. According to research by Hedrick et al., using a bifunctional catalyst combining (thio)urea and a tertiary amine yielded a 97% conversion rate, a molecular weight of 23 kg/mol, and a narrow polydispersity index of 1.05, albeit over an extended 48 h reaction time. The bifunctional catalyst using (thio)urea demonstrated excellent selectivity, with minimal racemization and transesterification even after the extended reaction time [53]. Kiesewetter’s research group elucidated why structurally similar alkylamine co-catalysts exhibit different activities and selectivities in the organocatalytic ROP of LLA using a thiourea/alkylamine dual-catalyst system. A novel hydrogen-bonding catalytic mechanism was proposed, where thiourea and alkylamine weakly bind in solution to form the active site. The reaction rate dependence and inhibition status vary depending on the base structure and its interaction mode with thiourea. Accordingly, new co-catalyst combinations effective for LLA ROP have been derived [54]. Thus, research on (thio)urea has largely focused on changing the type of base used. Systems combining stronger bases such as DBU, MTBD, phosphazene, and others have been integrated with highly electron-withdrawing diaryl(thio)ureas, leading to reports of highly active binary catalysts capable of producing PLLA with high conversion rates, predictable *M*_n_ values around 1.02, and narrow MWD within seconds to minutes near room temperature [55]. Within this trend, thiourea-based catalysts differ from approaches such as DBU or TBD, which rely on a single strong base. Instead, they differentiate by precisely controlling the arrangement of monomers and initiators through hydrogen bonding networks to suppress side reactions such as transesterification. Recently, research has expanded to introduce chiral thiourea [56,57] or bis(thiourea) [58] frameworks, aiming to achieve stereoselective ROP of rac-lactide and even tacticity control.

#### 2.2.3. ROP Using Enzyme Catalysts

Enzyme catalysts are biological catalysts that exhibit high substrate recognition and reaction selectivity in nature and have consistently drawn attention as catalysts for bio-based polymer synthesis (Figure 2b). Particularly when applied to ROP of cyclic esters such as lactide, they offer advantages such as high regioselectivity, enantioselectivity, chemoselectivity, and sophisticated stereoregulation, minimizing byproduct formation. They also enable polymerization under relatively mild reaction conditions regarding temperature, solvent, pressure, and pH [59]. However, enzyme catalysts are heat-sensitive, requiring low polymerization temperatures. This necessitates long reaction times to achieve high conversion rates and often limits polymer yield and molecular weight [60]. Therefore, research on lactide ROP using enzyme catalysts has recently shown a relative decline compared to studies using metal or organic catalysts. Nevertheless, enzyme catalysts remain an important research target due to their lack of residual metal issues and their combination of excellent selectivity and environmental friendliness. They are particularly recognized as a complementary platform to metal and organic catalysts when specific isomer selectivity or unique structural control is required. This paper introduces studies on ROP of lactides using representative enzyme catalysts such as *Candida antarctica* lipase B (CAL-B), *Burkholderia cepacia*-derived lipase (BCL), and porcine pancreatic lipase (PPL).

One representative enzymatic mechanism is the enzymatic ROP (eROP) of lactide using CAL-B. Early research by the Matsumura group compared various lipases, reporting that acrylate resin-immobilized CAL-B (Novozym 435) showed little to no activity or very low reactivity in bulk ROP of D,L-lactide, while other lipases exhibited higher activity [61]. This study first suggested that CAL-B’s substrate selectivity is biased toward D-lactide. Yoshida et al. directly polymerized L,L-lactide at 100 °C under bulk conditions using Novozym 435, yielding PLLA with a relatively low molecular weight (*M*_w_ = 2.4 kg/mol) and broad distribution. However, they demonstrated that the molecular weight can be gradually increased by adjusting reaction time and enzyme loading, providing a starting point for PLLA synthesis using immobilized CAL-B [62]. Moeller et al. precisely investigated the stereoselectivity and reactivity of PDLA while performing eROP using Novozym 435 on D,D-lactide under bulk and solvent conditions. They clearly demonstrated that CAL-B exhibits higher selectivity for the D-isomer compared to the L-isomer [63]. Further research has progressed toward controlling the reaction environment, with similar studies reporting the introduction of ionic liquids (ILs). The Yoshizawa group reported that when polymerizing LLA with CAL-B in various ILs, the polymer recovery yield tended to decrease as the solubility of PLLA in the IL increased. Simultaneously, they demonstrated that specific IL combinations could yield PLLA with a molecular weight of approximately 55,000 g/mol, emphasizing that solvent environment design is a key factor for obtaining high molecular weight PLA in CAL-B eROP [64]. María et al. synthesized hyperbranched PLLA by conducting eROP in a [C4MIM][PF_6_] IL medium. They also confirmed that the degree of branching could be controlled depending on the reaction conditions, demonstrating that CAL-B is an enzyme-catalyzed platform that can also be utilized for controlling branched structures and polymer topology [65]. In summary, lactide eROP using CAL-B (including Novozym 435) has expanded its research scope from the initial low molecular weight and broad distribution phase in bulk systems to elucidating substrate and stereoselectivity, optimizing reaction media, and controlling molecular weight and branching structure. Thus, it has evolved into an enzyme-catalyzed platform aiming for PLA synthesis under metal-free conditions, enabling high molecular weight, isomer selectivity, and structural control.

Another representative enzyme catalyst is *Burkholderia cepacia* lipase (BCL). The Matsumura group compared several lipases in bulk eROP of D,L-, L,L-, and D,D-lactide in bulk eROP. They reported that while Novozym 435 showed almost no activity toward D,L-lactide, BCL exhibited high activity in the 80–130 °C range, forming PLA with a maximum *M*_w_ = 126 kg/mol and yields of 10–20% based on D,L-lactide [66]. In subsequent studies, the Matsumura group analyzed the same system more precisely, reporting that reaction temperature and enzyme concentration significantly influenced polymerization rate and molecular weight during bulk ROP of D,L-, L,L-, and D,D-lactide using BCL. Notably, lactide polymerizability and the *M*_w_ of the resulting polymer significantly improved under low enzyme concentration conditions [61]. Thus, early studies on BCL focused on optimizing reaction conditions, while subsequent work aimed to reduce the reaction time of the enzyme catalyst by introducing additives. Avérous et al. compared BCL with the addition of the non-protonic amine base triethylamine (TEA) and investigated the eROP characteristics of L- and D-lactide. They found selectivity was observed only for LLA. At this point, the addition of TEA to lactide eROP significantly shortened the reaction time to approximately 48 h. However, a drawback is that, despite nearly complete conversion with both enzymes, *M*_n_ remained at low molecular weight levels of hundreds to thousands of g/mol [67]. In short, BCL is an enzyme catalyst capable of synthesizing relatively high molecular weight PLA via lactide eROP in bulk and various environments. It has evolved into a platform where the molecular weight and stereostructure of PLA can be tuned to some extent by controlling reaction temperature, moisture, additives, etc.

Porcine pancreatic lipase (PPL) is also one of the enzyme catalysts applied to lactide ROP in early studies. Matsumura et al. conducted lactide eROP using PPL as a catalyst alongside BCL and CAL-B, establishing PPL’s relative position as an enzyme exhibiting higher affinity for lactide than CAL-B but lower activity than BCL [61]. Afterward, the Matsumura research group used PPL to perform the ring-opening copolymerization of lactide/trimethylene carbonate, synthesizing poly(lactide-co-trimethylene carbonate) with a *M*_w_ of approximately 21 kg/mol [68]. They demonstrated that flexibility could be controlled by adjusting the composition, thereby extending the role of PPL to the design of PLA-based copolymers. However, subsequent comparative studies concluded that PPL exhibits lower activity and a more limited molecular weight range compared to *Pseudomonas cepacia* lipase or CAL-B. Therefore, despite its low cost, the use of PPL in ROP has been declining.

Enzyme catalysts have been commonly used for lactide eROP, but they often necessitate long reaction times, from hours to days. Additionally, under non-optimized conditions, the *M*_n_ is generally low, and yields are often lower compared to metal/organic catalyst systems. Despite various process optimization strategies proposed to overcome these limitations—such as systems using ILs or mixed catalyst systems incorporating auxiliary bases (TEA)—disadvantages such as high costs, significant enzyme consumption, and limitations in producing polymers with relatively high molecular weights persist. Consequently, related research is trending downward [69].

#### 2.2.4. Other Catalysts

In addition to the catalysts described earlier, various catalysts exist for lactide ROP, including metal-free Lewis acids, classical Lewis adducts (CLAs), and frustrated Lewis pairs (FLPs) (Figure 2b). This section focuses on summarizing systems based on metal-free Lewis acids [70], CLAs [71,72], and FLPs, excluding metal catalysts, organic catalysts, and enzyme catalysts.

Initially, most Lewis acids used in ROP were metal-based. However, concerns about residual metals and stricter regulations have increased interest in metal-free Lewis acids. Consequently, strong Brønsted acids, including HCl·Et_2_O [73] and trifluoromethanesulfonic acid [74], were introduced into lactide ROP, achieving high activity and relatively good molecular weight control. However, their strong corrosiveness and limitations in safety/handling were simultaneously noted. Against this backdrop, the Zhu research group introduced selenonium salts as metal-free Lewis acid catalysts to realize living cationic ROP of ε-caprolactone (ε-CL), δ-valerolactone, trimethylene carbonate (TMC), and LLA [70]. They designed the selenonium cation to adjust Lewis acidity by combining different anions such as BF_4_^−^, PF_6_^−^, and SbF_6_^−^. When operated with alcohol initiators or trace amounts of water, the mechanism activated the carbonyl group of cyclic esters to induce polymerization. For LLA, the selenonium/SbF_6_^−^ combination achieved a high conversion rate exceeding 90% at 110 °C. However, a significant discrepancy between the theoretical value and the molecular weight measured by SEC suggested the coexistence of transesterification and water-initiated species. Introducing a weaker Lewis acid by substituting one phenyl group of the selenonium with a propyl group reduced the polymerization rate but nearly eliminated side reactions. This indicates that the balance between activity and side reactions can be engineered by tuning the Lewis acidity through the structure of the selenonium salt and anion combination. Metal-free Lewis acid-based systems are evaluated as potential catalytic platforms for producing high-molecular-weight, low-dispersion polyesters in the synthesis of PLA and other aliphatic polyesters, addressing concerns about residual metal toxicity.

Meanwhile, research on CLA catalysts incorporating the Lewis pair concept in lactide ROP primarily began with systems using Zn(C_6_F_5_)_2_ as the Lewis acid. The Bourissou group reported that using Zn(C_6_F_5_)_2_ as the Lewis acid and phosphine/amine-based bases as Lewis bases, a strongly bound neutral adduct formed between the two components could promote the ROP of LLA and ε-CL, yielding well-controlled high-molecular-weight cyclic PLA and cyclic copolyesters [75]. In this mechanism, Zn(C_6_F_5_)_2_ formed a coordination bond with the monomer, while the amine/phosphine base acted in a nucleophilic manner on the monomer or initiator to generate an alkoxide species, and polymerization proceeded via a typical coordination–insertion process. Additionally, the Li group combined Zn(C_6_F_5_)_2_ with various organic superbases and intensively analyzed the classical Zn(C_6_F_5_)_2_/2DMAP system. They performed lactide ROP using Zn(C_6_F_5_)_2_/2DMAP CLAs in solvents with different temperatures and dielectric constants [71]. They confirmed that as the temperature increased or the solvent dielectric constant grew, the interaction between the Lewis acid and base weakened, leading to an increase in chain initiation rate. However, the Zn(C_6_F_5_)_2_/2DMAP series generally exhibited low activity and significant temperature dependence, suggesting the need for more practical catalyst design. Accordingly, the Li research team developed the ZnR_2_/2DMAP series, introducing Zn(C_6_F_5_)_2_ and ZnEt_2_ as Lewis acids with phenyl or ethyl substituents instead of perfluorophenyl [72]. The interaction between ZnEt_2_ and DMAP was relatively weaker than that in Zn(C_6_F_5_)_2_/2DMAP, allowing the Lewis pair to dissociate more readily. This facilitated the recovery of ZnEt_2_’s Lewis acidity, resulting in high initiation efficiency and catalytic activity. Indeed, the ZnEt_2_/2DMAP system was reported to rapidly ring-opening polymerize lactide at relatively mild temperatures (25–80 °C) in solvents such as toluene or 2-methyltetrahydrofuran, enabling the synthesis of PLA with high conversion rates and relatively narrow MWD in the 1.2–1.4 range. Thus, Zn(C_6_F_5_)_2_/2DMAP, ZnEt_2_/2DMAP, Zn(C_6_F_5_)_2_/NHC, and other Zn-based CLAs catalysts provide a platform for precisely controlling operating temperature, polymerization rate, topology, MWD, and chain-terminating functional groups by adjusting Lewis acid–base binding strength and ligand structure. This has established them as important reference points for subsequent Lewis pair system design.

To overcome the limitations of CLA systems and expand the potential of Lewis pairs, the concept of FLPs was introduced in lactide ROP [71]. In these FLP systems, a strong Lewis acid electronically activates the lactide carbonyl, while a bulky base attacks the carbonyl carbon in a nucleophilic manner to form a zwitterionic active species, enabling polymerization. This process typically yields well-controlled cyclic PLA. The research team led by Li employed Zn(C_6_F_5_)_2_ as the Lewis acid and utilized various organic superbases as Lewis bases, including DMAP, NHC, DBU, and MTBD. The results revealed that the sterically small DMAP strongly coordinates to the Zn center, forming a classical Zn(C_6_F_5_)_2_/2DMAP adduct. In contrast, the bulky DBU and MTBD cannot coordinate directly to the Zn center and instead attack the para position of the phenyl ring, forming an FLPs structure where Zn(C_6_F_5_)_2_ and the base are only partially bonded. Zn(C_6_F_5_)_2_/DBU and Zn(C_6_F_5_)_2_/MTBD FLPs were reported to rapidly ROP of LLA under relatively mild conditions (40–80 °C), yielding PLA with high molecular weight and a narrow MWD of 1.12–1.29. This is a representative example demonstrating that FLPs’ design can achieve high activity and good controllability, which are difficult to obtain with Zn/DMAP CLAs. In summary, FLPs systems such as Zn(C_6_F_5_)_2_/DBU and Zn(C_6_F_5_)_2_/MTBD present a catalyst design strategy that simultaneously achieves high activity, mild reaction conditions, cyclic PLA formation, and excellent molecular weight control by appropriately utilizing partially frustrated bonding states.

Furthermore, recent studies have actively explored ROP using metal-free Lewis pairs composed of p-block Lewis acids such as B(C_6_F_5_)_3_ [76] and triethylborane (TEB) [77] combined with organic bases. These systems provide benefits like high activity, mild conditions, and unique structural control of FLPs without metal residues. However, they are mainly limited to fundamental research and specialized polymer design due to air/moisture sensitivity, a narrow range of monomers, and the cost and handling issues of borane-based catalysts.

Catalyst stability under elevated temperatures and bulk polymerization conditions is a critical factor for practical applicability. In bulk systems, prolonged reactions at high temperatures can promote side reactions such as transesterification or back-biting, depending on catalyst type. Robust metal-based catalysts, such as Sn-based systems, generally retain activity under these conditions but often exhibit increased side reactions over extended reaction times, whereas many Zn- and Al-based catalysts require narrower processing windows to maintain molecular weight control and stereochemical fidelity. By contrast, organocatalysts and metal-free Lewis pair systems typically enable polymerization under milder conditions but often suffer from limited thermal stability and reduced tolerance to prolonged bulk operation. Collectively, these differences highlight time-dependent stability under bulk and high-temperature conditions as a key distinction among catalyst families rather than a universal property.

To facilitate comparison among these polymerization routes and catalyst systems, the key characteristics reported in the literature—including reaction conditions, achievable molecular weight, stereochemical control, scalability, and environmental or regulatory considerations—are summarized in Table 1. As summarized in Table 1, Sn(Oct)_2_-based systems remain dominant in industrial PLA production due to their robustness and high productivity; however, concerns regarding residual tin content and regulatory restrictions limit their applicability in biomedical and food-contact applications. Zn- and Al-based catalysts offer improved stereochemical control and reduced toxicity, but their sensitivity to moisture and narrower processing windows often hinder large-scale implementation. Metal-free organocatalysts and Lewis pair systems further expand the catalyst landscape by enabling polymerization under mild conditions with enhanced control over polymer topology and stereoregularity, albeit at the expense of catalytic activity and scalability. These comparisons highlight that no single catalyst system is universally optimal, and that catalyst selection should be guided by application-specific requirements rather than polymerization efficiency alone.

## 3. Tunable Physical and Biodegradable Properties of PLA

**Figure 3:**
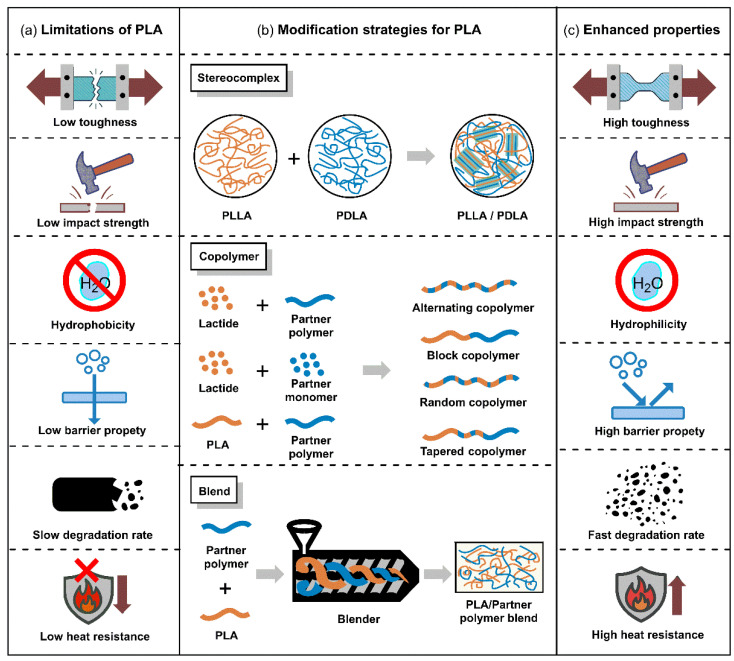
(**a**–**c**) Limitations of PLA and enhanced properties achieved through modification.

PLA is utilized in various industrial fields such as packaging materials, textiles, agriculture, medicine, and pharmaceuticals due to its excellent biocompatibility, biodegradability, and outstanding mechanical and optical properties. However, factors such as low toughness and impact strength, limited elongation and heat deflection temperature, slow crystallization rate, and slow biodegradation rate act as limiting factors for the widespread commercialization of PLA (Figure 3a). Thus, it is essential to define the target final properties and choose suitable structural design strategies, including SC formation, copolymerization, and blending [88]. This chapter summarizes how the mechanical and thermal properties, as well as the biodegradability, of conventional PLA can be controlled through stereocomplex PLA (scPLA) structures, PLA blend systems, and PLA copolymer design.

### 3.1. SC PLA

Stereocomplex PLA (scPLA) is a special crystalline structure formed when poly(L-lactide) (PLLA) and poly(D-lactide) (PDLA), which are enantiomers, undergo simultaneous crystallization during the crystallization process (Figure 3a). *T*_m_ of scPLA crystals is approximately 210–230 °C, generally more than 50 °C higher than the *T*_m_ of PLLA or PDLA homocrystal (HC) [89]. Due to its high *T*_m_ and excellent thermal stability, scPLA is particularly noteworthy in applications requiring high heat resistance, thermal stability, and enhanced mechanical strength.

**Figure 4:**
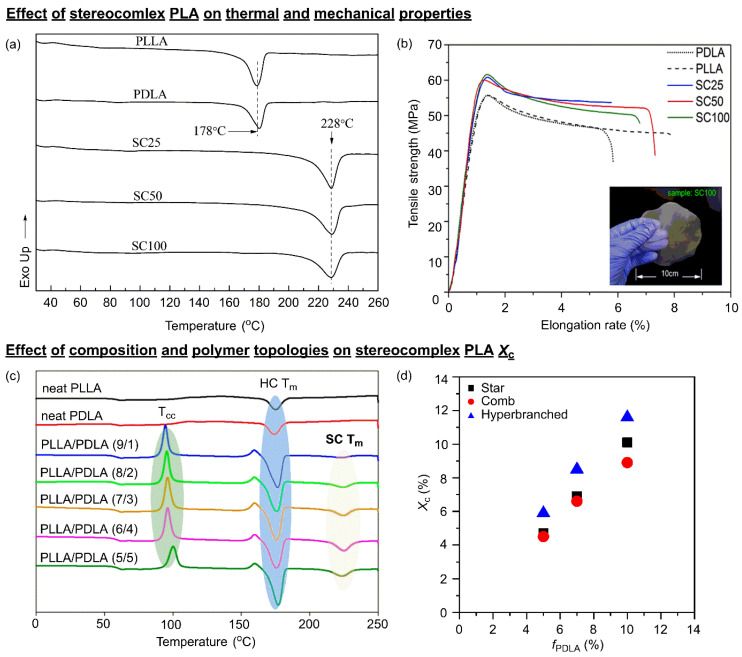
(**a**,**b**) Correlation between PLA stereocomplex and physical properties. (**c**,**d**) Effect of poly(L-lactide) (PLLA)–poly(D-lactide) (PDLA) composition and polymer topologies on crystallinity. (**a**) Reprinted from [93] under CC-BY 4.0 License. (**b**) Reprinted from [94] under CC-BY 4.0 License.

The Ikada group was the first to report that stereocomplex (SC) crystals form when PLLA and PDLA are mixed. They established that the *T*_m_ of scPLA crystals is approximately 50 °C higher than that of the homopolymer crystal and that thermal stability improves due to the densification of the crystal structure [90]. Differential scanning calorimetry (DSC) measurements of scPLA powder obtained from PLLA/PDLA mixtures at a 1:1 ratio and concentrations of 25, 50, and 100 mg/mL showed identical *T*_m_ peaks for scPLA at all concentrations in Figure 4a. This indicates that SC crystals exhibit distinct thermal behavior compared to HCs and supports that the enhanced mechanical performance of scPLA originates from its higher crystallinity and more stable crystal structure. A study comparing the mechanical properties of a PDLA/PLLA 1:1 blend and neat PLLA reported that the “physical cross-link” effect due to interchain interactions from SC formation increases Young’s modulus and stiffness but is accompanied by reduced ductility and increased brittleness [91]. This phenomenon can also be confirmed in experiments measuring actual stress–strain curves (Figure 4b). Furthermore, subsequent studies have confirmed that the crystalline structure varies depending on the composition of the homopolymer, revealing that crystallinity is particularly high when using low-molecular-weight enantiomers [92].

Early scPLA studies identified significant challenges, including high process and composition sensitivity: achieving complete SC formation in PLLA/PDLA blends required a PLLA:PDLA = 1:1 composition, and SC crystal formation was limited in high-molecular-weight PLLA/PDLA blends. Consequently, various strategies were proposed to improve scPLA synthesis routes. Coates et al. synthesized stereoblock PLA by arranging L/D blocks within a single chain via stereoselective ROP using a metal catalyst and precisely analyzed the SC formation behavior and thermal properties as a function of the block length within the chain [38]. Kimura et al. demonstrated that scPLA with higher *T*_m_ and controllable molecular weight could be obtained by polymerizing PDLA and PLLA in an SC form via a melt-solid polycondensation [86,87]. However, this process has limitations for direct application in medical or food fields due to toxic and incomplete catalyst removal after the reaction.

Furthermore, in linear PDLA/PLLA blend scPLA, the competition with HC formation and phase separation remains a persistent issue. Therefore, Satoh et al. designed a stereo-miktogram star structure with both PLLA and PDLA arms within a single molecule [95]. This strategy induced the formation of an SC within the molecule itself. This approach was reported to offer several advantages: reduced intermolecular mixing dependency, mitigation of competition with homocrystallization, and the potential to alleviate residual metal issues by utilizing organic catalyst-based ROP.

The high *T*_m_ (≈210–230 °C), low recrystallization temperature (*T*_cc_), and increased stiffness and modulus observed in scPLA are attributed to microstructural changes such as interchain interdigitation and hydrogen bonding, increased lamella thickness, and enhanced nucleation density. In short, the thermal behavior and mechanical properties of PLA depend on the amount of stereoregularity and crystallinity (*X*_c_). *X*_c_, a key factor determining SC thermal characteristics, varies with the initial PLLA/PDLA molecular weight, composition ratio, and topology, as studied by the Ikada group [96]. Generally, under identical conditions, the crystallinity of SC decreases as the molecular weight of the PLLA/PDLA racemic blend increases. This occurs because higher molecular weights reduce the probability of chain interaction in the melt state, and chain entanglement hinders SC nucleation. Experiments blending PDLA with molecular weights ranging from 7.5 to 290 kg/mol into actual PLLA revealed that the competitive relationship between SC and homo crystals varies depending on the PDLA molecular weight [97]. Furuhashi et al. confirmed that SC formation can be promoted when the molecular weight of either enantiomer is relatively small [98].

The ratio of PLLA to PDLA also significantly influences the degree of SC crystallization. While an ideal ratio close to 1:1 is most favorable for SC formation, as Figure 4c appears [94]. However, numerous experiments have reported that even a small amount of PDLA acts as an effective nucleation site for SC crystals within the PLLA matrix, significantly altering the overall crystallization behavior. Some studies have demonstrated that adding just 2 wt% PDLA forms an SC crystal network in the melt state. This network then acts as a strong heterogeneous nucleation site during the PLLA crystallization process, simultaneously controlling both the crystallization rate and the crystal structure [99,100].

Additionally, molecular topology directly influences SC formation and crystallinity. Dubois et al. found that scPLA blended with linear PLLA, incorporating star, comb, and hyperbranched PDLA, showed increased SC crystallinity with more PDLA branches and higher nucleating site density [101]. Figure 4d reveals that the SC crystallinity increases with the degree of branching, being highest for hyperbranched PDLA, followed by comb-type and then star-type PDLA. However, excessively high branch density or overly short branch lengths can inhibit SC formation since PLLA/PDLA chains cannot sufficiently interpenetrate [101]. The individual branch length is also a crucial factor determining *X*_c_. As branch length increases, molecular mobility decreases, leading to a tendency for *X*_c_ to increase [95]. Additionally, when blending nearly amorphous linear PLA with non-linear PLA, the crystallization rate is fastest for hyperbranched, followed by star, and then comb structures.

scPLA formation is an effective strategy that significantly enhances PLA crystallinity, simultaneously increasing its *T*_m_, heat resistance, and mechanical strength [90]. However, high crystallinity tends to reduce biodegradability, a key advantage of PLA, and challenges exist for commercialization, including the cost of high-purity PLLA/PDLA raw materials, sensitivity within processing, and increased film brittleness. Instead, the practical applicability of scPLA is confined to applications where high heat resistance, dimensional stability, and long-term mechanical integrity are prioritized over rapid biodegradation and low-cost processing. Typical examples include hot-fill food packaging, heat-resistant disposable products, and durable fiber or molded components with extended service lifetimes. In contrast, scPLA is less suitable for short-lived products or applications requiring fast environmental degradation, such as agricultural mulch films or compostable packaging designed for rapid turnover.

Accordingly, the effective utilization of scPLA requires clearly defined application boundaries and, in many cases, complementary material design strategies—such as blending or copolymerization—to balance performance gains with processability and degradation behavior. (Figure 3c).

### 3.2. PLA Blend

PLA blends have been extensively studied and applied in various fields, including packaging materials, due to their resistance to stresses encountered during storage and transportation and their excellent biocompatibility. However, PLA has several inherent limitations, such as low toughness and impact strength, and numerous blending strategies have been proposed to overcome these limitations (Figure 3b). The direction of property enhancement depends on the type of compatibilizer and polymers in the blend, with significant effort directed at using copolymer-based compatibilizers and plasticizers to improve blend compatibility. Blending PLA with flexible polymers such as polycaprolactone (PCL), poly(butylene succinate) (PBS), and poly(butylene adipate-co-terephthalate) (PBAT) is advantageous for blend partners because it improves ductility, toughness, and processing stability [102,103,104]. In contrast, poly(hydroxyalkanoate) (PHA) and poly(hydroxybutyrate) (PHB) possess higher crystallinity than PLA and are particularly effective in enhancing gas barrier properties [88,105]. Considering the broad range of potential blend partners, it is therefore crucial to define the target application and required properties. In this report, we focus on how representative blend partners such as PCL, PHB, and PBAT influence the biodegradability, barrier performance, mechanical properties, and processability of PLA.

#### 3.2.1. PLA/PCL Blend

PCL is a semicrystalline, biodegradable polyester with a *T*_g_ of about −60 °C and very high elongation and flexibility and has been the most extensively investigated blend component for toughening brittle PLA [106]. Early studies focused on the fact that PCL behaves like a polymeric plasticizer within the PLA matrix, greatly increasing elongation at break and impact strength while maintaining the stiffness of PLA [107,108]. However, PLA and PCL are thermodynamically poorly compatible and generally tend to phase separate [108]; the mechanical behavior strongly depends on the size and distribution of the dispersed PCL domains and on the degree of interfacial adhesion [109]. In PLA/PCL blends with coarse phase separation and weak interfacial bonding, simply increasing PCL content does not effectively activate energy-dissipation mechanisms, leading to a higher risk of premature fracture. Simple physical blending has clear limitations in simultaneously improving toughness and ductility while maintaining tensile strength and modulus.

Thus, subsequent studies have actively introduced compatibilization and morphology-control strategies to develop PLA/PCL blends into practical materials. Representative approaches include pre-synthesizing or in situ forming block/graft PCL–PLA copolymers that selectively localize at the interface [110]; introducing reactive chain extenders or compatibilizers that create chemical bonds or strong interactions at the PLA–PCL interface [111]; incorporating nanofillers to simultaneously control crystallization behavior and dispersion morphology [112]; and tailoring end groups prior to blending [113]. It has been reported that when the size of PCL domains is finely controlled and interfacial adhesion is enhanced, the elongation at break and impact strength can be increased by up to several tens of times compared to neat PLA, while the reductions in modulus and tensile strength can be kept to a minimum.

**Figure 5:**
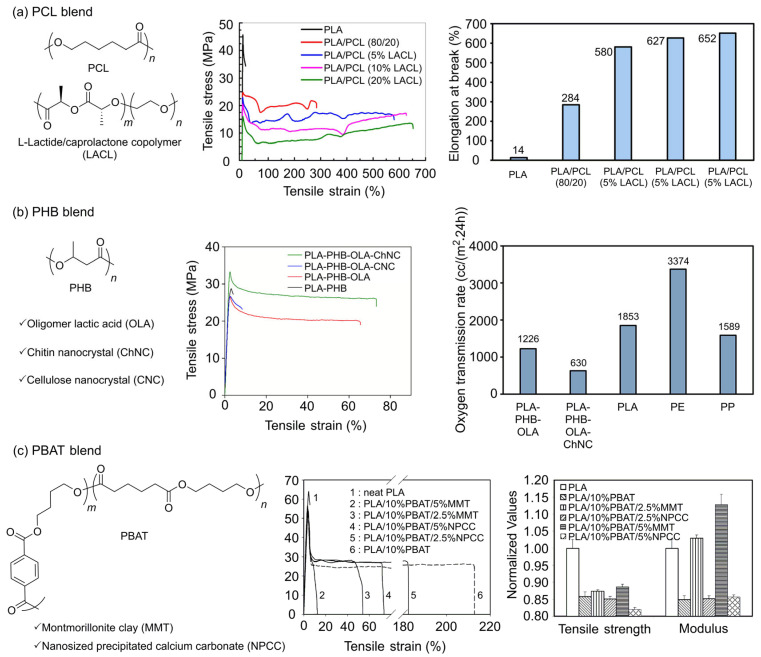
Correlation between (**a**) PLA/PCL, (**b**) PHB, (**c**) PBAT blend, and physical properties. (**a**) Adapted with permission from Ref. [110]. Copyright 2015 American Chemical Society. (**b**) Adapted from [105] under CC-BY 4.0 License. (**c**) Reprinted with permission from Ref. [114]. Copyright 2009 American Chemical Society.

Turng et al. introduced an LLA/caprolactone copolymer (LACL) as a compatibilizer into a phase-separated PLA/PCL (80/20 wt%) blend and quantitatively analyzed the effect of random copolymer compatibilization [110]. In ternary PLA/PCL/LACL systems, adding about 5 wt% LACL reduced the size of dispersed PCL domains and smoothed the interfaces, leading to a pronounced improvement in tensile deformation behavior (Figure 5a). In fact, the blend containing 5 wt% LACL exhibited a significantly higher elongation at break than neat PLA and the uncompatibilized PLA/PCL blend, while the decreases in tensile strength and modulus were relatively small, resulting in an improved balance between stiffness and toughness. Thus, LACL represents a typical example of a random copolymer compatibilizer that can simultaneously achieve morphological compatibilization (particle miniaturization), mechanical toughening, and modulation of PLA crystallization behavior. This study clearly highlights the importance of tuning microstructure via comonomer composition and molecular weight in the design of PLA/PCL blends.

Recent efforts have advanced the use of PLA/PCL blends beyond mechanical reinforcement to high-performance bio-blend platforms, allowing for simultaneous engineering of biodegradability, biocompatibility, surface functionality, and degradation rate. Kwak et al. synthesized a bio-toughener, highly branched poly(ε-CL)-grafted cellulose (CghbP), in which highly branched PCL chains are grafted onto a cellulose backbone, and proposed a strategy in which a small amount (5–10 wt%) of CghbP is incorporated into a PLA matrix [115]. PLA/CghbP blends exhibited partial compatibilization and a finely dispersed morphology, which led to more ordered PLA crystalline structures and markedly enhanced toughness. In particular, specimens containing 5 wt% CghbP showed almost unchanged tensile strength compared to neat PLA, while their elongation at break and tensile toughness increased by about threefold, demonstrating highly efficient toughening. Hydrolysis and soil burial tests further confirmed that the introduction of CghbP preserves biodegradability at a level comparable to PLA. This suggests that polymeric bio-tougheners such as CghbP can be used in place of low-molecular-weight plasticizers to improve the mechanical properties of PLA while maintaining its biodegradability.

Monticelli et al. sought to simultaneously address the brittleness, lack of functional groups, and limited control over the degradation rate of PLA films by designing PLA/star-PCL (80/20 wt%) blend films in which 20 wt% of a 4-arm star-PCL (each arm = 2 kDa) with different terminal groups was added [113]. Unlike linear PCL, star-PCLs bearing –OH, –COOH, or pyrenyl end groups improved compatibility with PLA via multiple chain ends and unique aggregation structures. Thus, whereas neat PLA showed brittle behavior with a modulus of 2150 MPa and an elongation at break of 4%, PLA/star-PCL-OH and PLA/star-PCL-COOH films maintained a modulus in the range of 1500–1760 MPa while increasing elongation at break to about 37–47%, achieving a much better stiffness–toughness balance than blends with linear PCL. Moreover, a simple variation in the end groups allowed tuning of surface hydrophilicity and the adsorption capacity toward cationic dyes. In cutinase-catalyzed hydrolysis tests, commercial PLA/PCL-L blends were almost completely degraded within a short time, whereas star-PCL-based films showed only limited mass loss (around 20%) after 7 days, displaying a slower, more controllable degradation behavior similar to that of PLA. These results indicate that by tailoring the architecture and end groups of low-molecular-weight star-PCL, it is possible to tune the toughness, surface functionality, and degradation rate of PLA/PCL blends without additional compatibilizers.

In summary, research on PLA/PCL blends has evolved from early, simple toughening strategies that relied on the low *T*_g_ of PCL to compensate for the brittleness of PLA to blend designs where phase separation and interfacial structure are finely controlled using block/graft PLA–PCL copolymers, random copolymer compatibilizers such as LACL, reactive chain extenders, and nanofillers. Recently, polymeric bio-tougheners such as CghbP and engineered 4-arm star-PCL have advanced PLA/PCL systems into high-performance bio-blend platforms. These platforms enable the simultaneous tuning of mechanical properties, biodegradability, surface functionalities, and degradation rates without the need for low-molecular-weight plasticizers or extra compatibilizers (Figure 3c).

#### 3.2.2. PLA/PHB Blend

PHB is a bio-based, biodegradable polyester that exhibits high crystallinity and excellent oxygen barrier performance but suffers from poor thermal stability, limited processability, and pronounced brittleness [116,117]. In contrast, PLA shows better processability and stiffness than PHB, but its low crystallinity and insufficient oxygen/water vapor barrier properties are clear drawbacks. Accordingly, blending the two polymers to complement each other’s weaknesses has been attempted for many years [117,118]. Early studies indicated that PLA/PHB can show partial miscibility or phase separation based on molecular weight and blend composition. Adding approximately 20–25 wt% PHB into PLA results in finely dispersed PHB spherulites, significantly enhancing the crystallinity and oxygen barrier performance of PLA. However, at this stage, PLA/PHB blends exhibit high brittleness and low elongation for both components, along with poor processability due to PHB’s low thermal stability [119,120,121].

Subsequent developments focused on improving miscibility and processability. Numerous experimental studies have actively attempted to lower *T*_g_ and secure higher extensibility by incorporating various plasticizers and active additives into PLA/PHB blends. For example, the addition of polyester-type plasticizers such as Lapol 108 [122] or additives such as acetyl tributyl citrate (ATBC) [123] and oligomeric lactic acid (OLA) [105] has been shown to reduce the *T*_g_ of PLA/PHB blends by several tens of degrees Celsius and increase elongation at break, while simultaneously introducing a certain trade-off where strength and thermal stability are partially sacrificed.

Recent research has focused on identifying compositions for direct application in industrial processes such as film blowing and quantitatively designing structure–processing–property relationships. Oksman and co-workers prepared film-blown PLA–PHB–OLA–chitin nanocrystal (ChNC) films by introducing 4 wt% OLA as a plasticizer/compatibilizer into a PLA/PHB (75/25 wt%) matrix and further adding 1 wt% ChNC [105]. In the presence of OLA, ChNCs were uniformly dispersed at the nanoscale within the PLA/PHB matrix, stabilizing the film-blowing process and producing smooth films, and showed superior dispersion and reinforcing effects compared with more strongly aggregated cellulose nanocrystals (Figure 5b). The reference PLA/PHB blend film showed brittle behavior with a tensile strength of 26.8 MPa and an elongation at break of about 2%. In contrast, the PLA–PHB–OLA–ChNC film had a tensile strength of 36.8 MPa, a 71% elongation at break, and a significant increase in toughness, while maintaining the modulus. The oxygen permeability was reduced to about half that of PLA/PHB–OLA, achieving barrier properties competitive with commercial polyethylene (PE)/polypropylene (PP) films, whereas water vapor permeability and disintegration rate under composting conditions increased. Thus, this study proposed a strategy that simultaneously satisfies processability, mechanical performance, barrier properties, and compostability required for short-shelf-life food packaging.

Meanwhile, D’hooge et al. developed a reactive blending strategy aimed at simultaneously addressing the issues of elongation, stiffness, and biodegradability of PHB [124]. They blended deliberately thermally degraded PHB (dPHB) with PLA, introducing maleic anhydride (MA) and, when needed, dicumyl peroxide (DCP) as reactive components. Since thermally degraded dPHB possesses shorter chains and higher mobility than the original high-molecular-weight PHB, its compatibility with PLA is improved; simultaneously, MA and radical initiation induce PLA–dPHB graft structures, chain extension, and partial crosslinking, leading to the formation of a homogeneous, fine phase morphology. Unlike conventional PLA/PHB blends, where impact strength increases only slightly and elongation improvement is negligible, PLA/dPHB/MA (75/25 wt%, 7 phr MA) biocomposites were reported to retain high stiffness and strength (tensile modulus 1.8 GPa and yield strength 22.1 MPa) while showing a large increase in elongation at break to 131.3% and a significant enhancement in impact strength. This study demonstrates a modern design direction that simultaneously targets improved mechanical properties and circularity by recycling waste PHB—via thermal degradation—into a value-added raw material for reactive PLA/PHB blends.

In summary, research on PLA/PHB blends initially focused on clarifying composition- and molecular-weight-dependent miscibility and crystallization behavior. In particular, the introduction of around 25 wt% PHB has been shown to enhance the crystallinity and barrier performance of PLA. Subsequent work has largely aimed to mitigate the brittleness and poor processability of both polymers through the incorporation of plasticizers such as OLA and ATBC, reactive compatibilizers, and nanofillers. Recently, studies such as the ChNC nanocomposite films of Patel and Oksman and the dPHB–MA reactive blends of D’hooge have advanced toward quantitatively linking specific processing conditions (e.g., film blowing, reactive extrusion) with microstructure, mechanical properties, and degradation behavior, ultimately proposing formulations that can be directly implemented in real food-packaging and recycling contexts (Figure 3c).

#### 3.2.3. PLA/PBAT Blend

PBAT blends are representative biodegradable systems that can combine the high strength and stiffness of PLA with the excellent ductility and flexibility of PBAT [125]. Thus, initial studies on PLA/PBAT mainly focused on toughening brittle PLA by adding PBAT. However, the two polymers exhibit typical immiscible behavior and poor interfacial adhesion. Several reports indicate that increasing PBAT content significantly boosts elongation at break up to certain levels, while strength, modulus, and thermal stability decrease [126]. Thus, simple blending is insufficient to obtain a well-balanced set of properties. Zhang et al. investigated ternary PLA/PBAT nanocomposites early on, finding that PLA/PBAT/nanofiller blends with montmorillonite clay (MMT) show higher tensile strength and modulus compared to those with nanosized precipitated calcium carbonate (NPCC), though this comes with reduced elongation (Figure 5c) [114]. They further showed that introducing maleic-anhydride-grafted PLA (MA-g-PLA) as a compatibilizer improves nanoparticle dispersion and interfacial stability, thereby restoring and even increasing the elongation at break of the ternary composites. These early nanocomposite studies indicated that combining PBAT with nanofillers can significantly compensate for the low toughness and elongation of PLA, but that it remains challenging to simultaneously satisfy both strength and modulus.

Subsequent work in PLA/PBAT systems has centered on compatibilization strategies that finely tailor the size and morphology of the dispersed PBAT phase and the interfacial chemistry between the two phases. For example, Ji et al. showed that adding a small amount of PLA–PBAT–PLA triblock copolymer can effectively compatibilize PLA/PBAT blends, as the triblock copolymer penetrates into each homopolymer phase and forms a highly entangled interphase [127]. They designed PLA–poly(ethylene) glycol (PEG)–PLA triblock copolymers containing hydrophilic PEG segments to emulsify the interfacial layer and enhance interfacial adhesion, thereby increasing compatibilization efficiency [128]. Therefore, the interfacial structure of PLA/PBAT blends became more compact, and improvements in tensile strength and elongation at break were achieved.

Bai et al. designed a reactive modifier, epoxy-functional styrene-acrylic oligomer (ESA)-graft-PDLA, by grafting PDLA onto an ESA, and proposed its use as a compatibilizer for PLLA/PBAT blends [129]. During reactive melt blending, ESA-g-PDLA reacts with PBAT chains to form PBAT-g-PDLA copolymers at the interface, while the PDLA branches co-crystallize with PLLA matrix chains to generate SC crystallites localized at the interface. These SC crystallites act as highly effective compatibilizers, inducing much stronger interfacial adhesion and finer phase dispersion than conventional interfacial copolymers. Therefore, the addition of only 0.5 wt% ESA-g-PDLA increased the impact strength of a PLLA/PBAT (70/30 wt%) blend from 3.6 kJ/m^2^ to 39.6 kJ/m^2^, while the SC crystallites acted as nucleating agents for PLLA, significantly enhancing both the crystallization rate and degree of crystallinity. Furthermore, PLLA/PBAT (70/30) samples containing 1.5 wt% ESA-g-PDLA exhibited a high impact toughness of about 53.2 kJ/m^2^ and excellent heat resistance with a heat distortion temperature around 124 °C, while still maintaining a high tensile strength of approximately 48.9 MPa. These results demonstrate that compatibilization strategies based on interfacially localized SCs can simultaneously achieve a favorable balance of strength, toughness, and heat resistance. This method utilizes the location and quantity of SC crystallites as design parameters, offering interfacial design guidelines to improve mechanical properties and thermal resistance in other PLLA-based biodegradable blends.

PLA/PBAT blends have progressed beyond simple composition optimization to become an important system to be developed into next-generation biodegradable engineering plastics. By precisely designing interfacial copolymer formation, SC crystallization at the interface, and cooperative effects with nanofillers, it is possible to simultaneously satisfy requirements for strength, toughness, heat resistance, and processability (Figure 3c).

Taken together, the thermal and mechanical properties of PLA are strongly governed by crystallinity and stereochemical structure. Representative literature data indicate that neat PLA typically exhibits a glass transition temperature (*T*_g_) of approximately 55–60 °C and a melting temperature (*T*_m_) around 150–170 °C, while stereocomplex formation significantly increases *T*_m_ to values exceeding 220 °C. Mechanical performance, including tensile strength and elongation at break, varies widely depending on molecular architecture and modification strategy. To facilitate quantitative comparison across these studies, representative numerical values are summarized in Table 2. As summarized in Table 2, stereocomplex formation markedly increases the melting temperature of PLA, whereas blending strategies generally improve toughness at the expense of modulus and thermal resistance. These quantitative trends highlight the inherent trade-offs in structure–property relationships.

### 3.3. PLA Copolymer

PLA is a representative biodegradable polymer derived from renewable resources such as corn and sugarcane and has attracted considerable attention as a sustainable alternative to conventional petrochemical-based plastics owing to its excellent biocompatibility and mechanical strength [147]. However, PLA inherently has several drawbacks: high brittleness, low thermal stability, low heat distortion temperature, and high hydrophobicity, which limit its use in certain applications (Figure 3b) [148]. One of the most effective strategies to overcome these shortcomings and tailor material properties for specific end uses is copolymerization. Copolymerization involves chemically incorporating two or more different monomers into a single polymer chain, thereby enabling the introduction of new functionalities or the fine-tuning of existing properties that are difficult to achieve with the corresponding homopolymer [149]. Research on PLA copolymers has focused on controlling degradation rate, enhancing mechanical flexibility, and increasing hydrophilicity, expanding the application window of PLA through systematic design of comonomer structure and composition. In this report, we aim to provide a systematic analysis of major PLA-based copolymer systems that have been extensively studied to address these limitations—specifically those incorporating PCL, PEG, and polyglycolic acid (PGA)—with an emphasis on the properties imparted by each comonomer and the resulting application fields.

#### 3.3.1. PLA/PCL Copolymer

PCL is a typical ductile/tough biodegradable polyester with a *T*_g_ of about −60 °C and very high elongation at break and has been widely studied as a soft segment for toughening intrinsically brittle PLA [106]. Early studies synthesized random copolymers of LLA and ε-CL, PLLA–PCL–PLLA triblock copolymers, and PLLA–PCL multiblock copolymers, and demonstrated that the incorporation of PCL segments lowers the *T*_g_ and generates rubbery domains, significantly enhancing the elongation at break and impact strength of PLA [135]. These systems typically comprise a PCL-based soft phase and a PLA-based hard phase, and it has been shown that elasticity, stiffness, crystallinity, and degradation rate can be tuned by varying the PCL content and block length.

**Figure 6:**
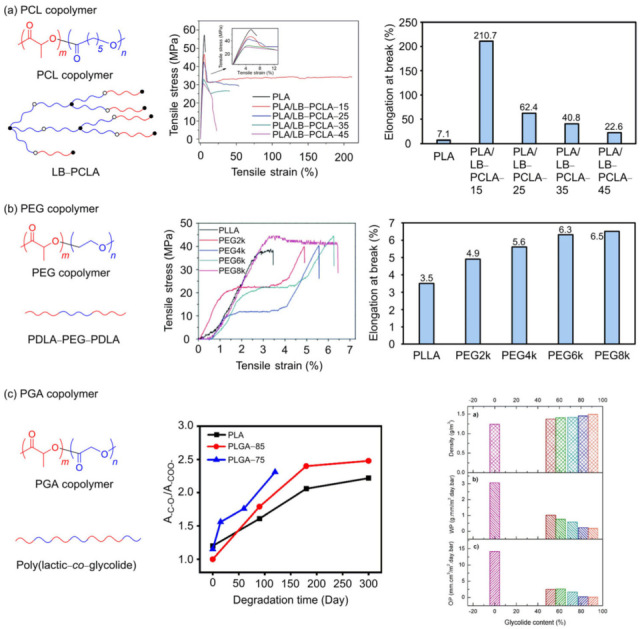
Schematic illustration of representative blending and modification strategies of PLA with biodegradable polymers, including poly(butylene adipate-co-terephthalate) (PBAT), poly(butylene succinate) (PBS), poly(ε-caprolactone) (PCL), and poly(3-hydroxybutyrate) (PHB), and their effects on toughness, flexibility, and processability. Panels (**a**–**c**) show representative examples of (**a**) PLA/PCL blending, (**b**) plasticization with polyethylene glycol (PEG), and (**c**) copolymerization with poly(glycolic acid) (PGA). (a) Adapted with permission from Ref. [150]. Copyright 2015 Royal Society of Chemistry. (b) Adapted with permission from Ref. [151]. Copyright 2020 Royal Society of Chemistry. (c) Reprinted from [130] under CC-BY 4.0 License. Reprinted from [152] under CC-BY-NC-ND 4.0 License. https://pubs.acs.org/doi/10.1021/acsapm.0c00315 accessed on 30 December 2025.

Subsequent work moved beyond simple random/block copolymers toward a more sophisticated chain-architecture design between PLA and PCL, with the aim of simultaneously improving mechanical performance and processability. Yang and co-workers proposed a strategy in which a long-chain-branched copolymer (LB-PCLA) composed of PLA and PCL is introduced to simultaneously enhance crystallinity, mechanical properties, and rheological behavior [150]. In this design, LB-PCLA is a branched PLA-b-PCL block copolymer synthesized from mono-hydroxyl-terminated PLA (PLA–OH) and a trifunctional PCL with three hydroxyl end groups (PCL–3OH), such that chemically bonded branch structures between PLA and PCL allow fine control over phase separation morphology and interfacial structure. Additionally, a PLA/LB-PCLA blend containing about 15 wt% LB-PCLA in the PLA matrix exhibited more than a 30-fold increase in elongation at break compared with neat PLA, with the fracture behavior shifting from brittle to ductile (Figure 6a). These differences in tensile strength and elongation at break reflect the influence of copolymer architecture, where block PLA/PCL structures promote phase-separated morphologies that enhance ductility, while random architectures tend to suppress crystallinity and reduce mechanical reinforcement.

Uyama et al. designed alternating multiblock copolymers in which PLA and PCL segments are arranged in an alternating fashion to address both the low toughness of PLA and its poor degradability in marine environments [136]. In this system, PCL-diol is first used as an initiator for ROP of LLA to form PLA–PCL–PLA triblock copolymers; these are then chain-extended with hexamethylene diisocyanate (HDI) in a second step to generate multiblock structures in which PLA and PCL segments alternate along the chain. By varying the feed amount of LLA, the PLA segment length (PLA 50–90 wt%) was tuned, and the resulting mechanical, thermal, and degradation properties were compared. For the composition containing 84 wt% PLA, the toughness increased dramatically from about 0.61 MJ/m^3^ for neat PLA to 83.4 MJ/m^3^, and the elongation at break rose from 2.26% to 803%. Furthermore, in the alternating multiblock architecture, the regular insertion of flexible PCL segments lowers *T*_g_ below room temperature and reduces crystallinity, leading to greater chain flexibility than in neat PLA and an accelerated hydrolysis rate in aqueous media. Therefore, this system has been proposed as a design platform capable of simultaneously improving mechanical performance and biodegradability.

Overall, PLA/PCL copolymers have established themselves as a representative family of biodegradable block copolymers in which the combination of PCL soft segments and PLA hard segments, together with their microstructure (random, block, multiblock, long-chain-branched), can be used as design parameters to finely balance *T*_g_, *T*_m_, crystallinity, toughness, and degradability. The design principles accumulated in this field can serve as generally applicable structural guidelines for the future development of high-performance, high-toughness bioplastics based on PLA (Figure 3c).

#### 3.3.2. PLA/PEG Copolymer

PEG is a polymer with flexible chains, high hydrophilicity, and excellent biocompatibility, and has been widely used as a comonomer to introduce soft segments into intrinsically brittle PLA to increase elongation at break and toughness and to impart moisture affinity [139]. In earlier studies, PLA/PEG copolymers were typically synthesized as PLA-co-PEG random copolymers or PLA–PEG–PLA triblock structures via melt polycondensation or ROP of lactide, and research mainly focused on summarizing how changes in PEG content and chain architecture led to decreased *T*_g_, increased elongation at break, and simultaneous increases in moisture affinity and hydrolysis rate [153,154,155].

Subsequently, Li et al. designed PDLA–PEG–PDLA triblock copolymer-based PDLA–PEG–PDLA/PLLA blends and investigated how PEG molecular weight (2, 4, 6, and 8 kDa) affects PLA SC formation, crystallization behavior, thermal stability, and tensile properties [151]. As the PEG molecular weight increased, both the onset decomposition temperature and the main decomposition peak temperature shifted to higher values, indicating that SC formation enhances the thermal stability of the blends. From a mechanical standpoint, whereas neat PLLA exhibited brittle behavior with a tensile strength of 38.4 MPa and an elongation at break of 3.5%, the blends containing PDLA–PEG–PDLA showed tensile strengths up to 44.5 MPa and elongations at break up to 6.5%. These results revealed a clear structure–property relationship where the SC crystals provide high strength and heat resistance, while the PEG segments contribute chain flexibility (Figure 6b).

To address the problem of extremely slow degradation of PLA in marine environments, Uyama et al. synthesized sequence-controlled PEG–PLA multiblock copolymers in which hydrophilic PEG is periodically inserted into PLA chains and compared how alternating versus random architectures and PLA segment length influence mechanical properties, water resistance, and degradation behavior in seawater and enzyme-containing media [140]. Alternating PEG–PLA multiblocks with sufficiently long PLA segments exhibited high regularity and semicrystallinity and showed excellent long-term water resistance in deionized water, maintaining molecular weight even after 6 months, while achieving tensile strengths up to 42.6 MPa and high toughness in both dry and wet states. In contrast, multiblocks with random architecture or shorter PLA segments were more amorphous and hydrophilic, which facilitated penetration of seawater and proteinase K solutions; these materials showed very rapid marine degradation, with a biodegradation degree of 72.63% and a weight loss of 71.5% over 28 days in the OECD 306 Biodegradability (BOD) test. Degradation in this system occurred primarily through preferential chain scission of PLA segments in the PEG–PLA blocks. It was proposed that adding PEG and the related hydrogen-bonding network enhances nucleophilic hydrolysis in seawater, leading to a significant drop in *M*_n_ and a strong BOD response in marine environments.

The same group further extended the PEG–PLA multiblock concept to the biomedical field by designing poly(LLA)-based shape-memory multiblock copolymers as candidate materials to replace conventional metallic esophageal stents [141]. They synthesized PLA–PEG–PLA triblocks via ROP of PLA from PEG diols and then chain-extended them with HDI to form physically crosslinked networks. In these shape-memory PEG–PLA multiblocks, adjusting the lengths of PEG and PLA segments allowed the *T*_g_ to be tuned in the range of 31.9–54.6 °C. In particular, the PEG4000/PLA1500 composition exhibited a dual (thermal/moisture-induced) shape memory effect at body temperature (37 °C), achieving a shape recovery ratio as high as 99.5% under thermal and water stimulation. These multiblocks also showed water uptake values of 41–328%, elongation at break of 140–1900%, and pH-dependent biodegradation behavior in simulated gastrointestinal environments. This combination of qualities indicates that such materials can provide adequate mechanical support and flexibility in the esophagus before gradually degrading and being eliminated in the intestine without the need for a secondary removal surgery.

Research on PLA/PEG copolymers has progressed from initial attempts to reduce PLA brittleness and enhance processability with PEG to advanced systems such as Li’s SC-based high-strength, high-heat-resistant PDLA–PEG–PDLA/PLLA blends and Uyama’s controlled PEG–PLA multiblocks for optimizing marine biodegradability and designing shape-memory networks. Therefore, PLA/PEG copolymers have progressed into precision-designed PLA-based bioplastic platforms spanning applications from marine-degradable packaging to bioresorbable esophageal stents (Figure 3c).

#### 3.3.3. PLA/PGA Copolymer

Poly(lactide-co-glycolide) (PLGA), a copolymer of PLA and PGA, was not originally conceived as a high-performance biodegradable polymer platform; rather, its development began with the design of synthetic absorbable sutures. Early work focused on applying PLA, PGA, and their copolymers as surgical suture materials and systematically investigating how the lactide/glycolide ratio, molecular weight, and crystallinity should be tuned to control hydrolysis rate [156,157]. Subsequent research actively extended PLA/PGA copolymers to drug-delivery micro- and nanoparticles [158,159], implants, and tissue-engineering scaffolds.

As the inherently low gas-barrier properties and slow environmental degradation of PLA emerged as critical limitations, efforts intensified to reinterpret PLA/PGA copolymers as high-barrier, environmentally friendly packaging materials. PGA exhibits crystallinity up to about 52% [143], a *T*_m_ of 220–225 °C [144], and oxygen barrier performance superior to that of PLA [145], but its very high *T*_m_ and brittleness make it difficult to process and use on its own. Consequently, glycolic acid (GA)-rich PLGA began to attract attention for combining the excellent barrier properties of PGA with the processability of PLA. Wan et al. synthesized PLGA with high G content, such as L/G = 25:75, via ROP at relatively low reaction temperatures and high conversion [146]. These compositions remain amorphous despite their high GA content, enabling the production of transparent products, and the authors clarified how reaction parameters must be adjusted to achieve both high molecular weight and high conversion when synthesizing GA-rich PLGA. Gruter et al., using GA derived from CO_2_ as a feedstock, prepared a series of PLGAs containing 50–91 mol% GA and systematically analyzed the correlations between GA content, thermal stability, and oxygen/water vapor barrier properties [152]. They found that increasing GA content enhances thermal stability and improves barrier performance toward both oxygen and water vapor, demonstrating that high-GA, high-barrier PLGAs can be used not only as medical materials but also as promising sustainable packaging candidates based on CO_2_-derived monomers (Figure 6c).

The application scope of PLGA has expanded beyond medical and packaging uses to explicitly consider degradation behavior in real environmental conditions. Zhang et al. prepared films of PLA, PGA, PLGA-85, and PLGA-75 (where PLGA-85 contains 85 mol% PLA) and immersed them in simulated marine environments for extended periods to compare and analyze their degradation behavior [130]. In all samples, ester-bond hydrolysis was identified as the common degradation mechanism, but the degradation rate followed the order PGA > PLGA-75 > PLGA-85 > PLA. They also quantitatively confirmed preferential hydrolysis of GA units during degradation and established a direct correlation between GA content and degradation rate (Figure 6c). By extending the discussion of PLGA degradation from primarily in vivo or composting conditions to marine environments, this study demonstrated that GA content, hydrophilicity, and crystallinity can be used as design parameters to control degradation kinetics when engineering high-barrier packaging materials or fibers with potential marine exposure.

Coates et al. further investigated how sequence control in PLA/PGA copolymers affects hydrolysis kinetics, bulk morphology, and thermal behavior, and showed that different microstructures exhibit unique degradation profiles [160]. Even at identical LA/GA compositions, a higher proportion of G–G linkages (G = glycolide) leads to faster hydrolysis than L–G or L–L linkages (L = lactide), because G–G bonds are more susceptible to hydrolytic cleavage. Alternating-sequence PLGAs, lacking G–G and L–L linkages, degrade more slowly and show linear mass loss with sustained drug-release profiles, unlike random 50/50 PLGA. These findings highlight that hydrolysis rate and release characteristics in PLGA systems can be finely tuned through sequence control.

PLA/PGA copolymers have evolved from their origins as suture-grade medical materials into platforms for precisely designing degradation times in drug delivery and tissue engineering. Subsequently, they have been further developed into high-barrier packaging materials that combine the outstanding gas barrier properties of PGA with the renewability of PLA and even into carbon-circular materials that utilize CO_2_-derived monomers. The main research focus for PLA/PGA copolymers will be to attain high barrier performance, adjustable degradation rates, and carbon circularity by controlling GA content, copolymer microstructure, and crystallinity (Figure 3c).

Beyond their impact on mechanical and barrier properties, the structural parameters discussed above—including crystallinity, copolymer composition, and microstructure—also play a decisive role in governing the biodegradation behavior of PLA-based materials. Modifications intended to enhance functional performance inevitably alter chain mobility, water accessibility, and susceptibility to hydrolytic or enzymatic attack, thereby influencing degradation kinetics under different environments. To clarify how these structure–property relationships translate into practical degradation behavior, representative quantitative data on PLA biodegradation under different environments are summarized in Table 3. As summarized in Table 3, PLA exhibits rapid degradation under industrial composting conditions, whereas degradation under soil and marine environments proceeds much more slowly, often over timescales of several months to years. Increased crystallinity, stereocomplex formation, and incorporation of PGA segments generally retard degradation by restricting chain mobility and water penetration, while blending with more flexible or hydrophilic components can accelerate molecular weight loss under specific conditions. In particular, under environmental conditions where degradation proceeds slowly, partial fragmentation of PLA-based materials prior to complete mineralization has been discussed in the literature, raising concerns about the potential persistence of microplastic-sized residues. Importantly, such effects are strongly dependent on test environment, temperature, exposure duration, and material design, and therefore should not be generalized without explicit experimental conditions. These observations demonstrate that biodegradation performance cannot be generalized without specifying both environmental conditions and material design parameters. Overall, the physical properties and biodegradation behavior of PLA are closely interconnected and strongly governed by molecular structure, crystallinity, and phase morphology. Strategies employed to enhance mechanical, thermal, or barrier performance—such as stereocomplex formation, copolymerization, and blending—inevitably influence degradation behavior under different environments. These interdependencies underscore the importance of considering structure–property–degradation relationships when designing PLA-based materials for specific applications and end-of-life scenarios.

## 4. Application of PLA

PLA is a representative bio-based thermoplastic polymer produced from renewable resources such as cornstarch and sugarcane. It has attracted considerable attention as a promising alternative to conventional petroleum-based plastics owing to its excellent transparency, mechanical strength, processability, biocompatibility, and biodegradability. In particular, its biodegradability under appropriate conditions offers significant advantages in mitigating plastic waste and carbon emissions, which has driven extensive research and development of PLA-based materials across diverse application fields, including food packaging, textiles, agricultural materials, and medical applications [163].

Despite these advantages, the relatively low heat deflection temperature, inherent brittleness, and limited oxygen and moisture barrier properties of PLA restrict its use in high-value packaging, heat-resistant fibers, agricultural films, and structural materials requiring long-term durability [131]. Accordingly, various modification strategies—such as stereocomplex formation, blending, and copolymerization—have been explored to overcome these limitations and to simultaneously achieve the mechanical, thermal, and barrier properties required for specific applications [131]. This chapter, therefore, summarizes the property requirements, corresponding structural design strategies, and recent research trends for representative application areas in which PLA is actively utilized, namely food packaging, fibers and clothing, and agricultural materials.

From an application perspective, different end-use scenarios impose distinct and sometimes competing requirements on thermal and mechanical performance. Representative ranges of key properties, together with corresponding material design strategies reported in the literature, are summarized in Table 4. As shown in Table 4, food packaging and heat-resistant packaging applications generally require elevated melting temperatures and dimensional stability, which can be achieved through stereocomplex formation or controlled crystallization. In contrast, applications demanding flexibility and toughness, such as fibers and agricultural films, benefit from blending or copolymerization strategies that increase elongation at break, often at the expense of modulus or thermal resistance. These comparisons highlight the application-specific nature of PLA material design.

### 4.1. Food Packaging

**Figure 7:**
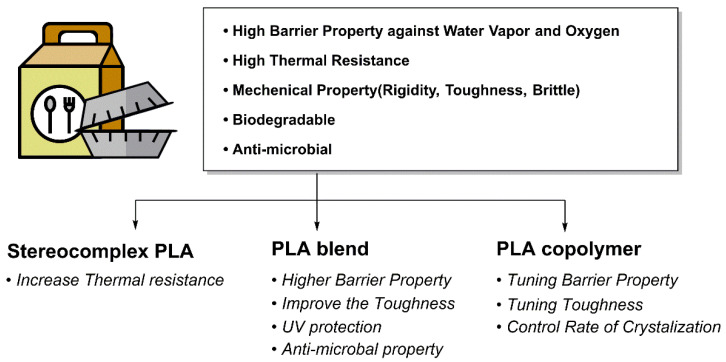
Required functional properties in food packaging and PLA-based strategies to achieve these requirements.

The primary purpose of food packaging materials is to prevent spoilage during distribution and storage while maintaining food quality and safety. Accordingly, packaging materials must exhibit effective barrier properties against oxygen and moisture, sufficient mechanical strength and impact resistance, heat resistance, chemical stability, and, in some cases, antimicrobial functionality (Figure 7). More recently, biodegradability has emerged as an additional key requirement to reduce environmental impact after use.

Among bio-based polymers, polylactic acid (PLA) has attracted particular attention as a food packaging material due to its transparency, processability, and biodegradability. PLA received approval for food-contact applications from major regulatory agencies, including the U.S. Food and Drug Administration (FDA) and the European Food Safety Authority (EFSA), which enabled its early commercialization as transparent packaging films in the late 1990s. As a result, PLA has been widely adopted in disposable food packaging applications such as containers, cups, trays, and films [199].

Despite these advantages, the relatively low heat deflection temperature, insufficient oxygen and moisture barrier properties, and inherent brittleness of PLA have limited its broader application in food packaging. From a cost perspective, PLA remains more expensive than commodity polyolefins such as polypropylene, although it is increasingly competitive with polyethylene terephthalate in packaging segments where transparency and sustainability considerations are prioritized. Consequently, early research efforts focused on improving toughness and heat resistance through the incorporation of plasticizers (e.g., polyethylene glycol or oligomeric lactic acid) or by controlling crystalline structure via thermal treatment.

Subsequently, research intensified on utilizing the scPLA structure to achieve the high barrier properties required for food packaging. It has been established that the melting point of scPLA is approximately 50 °C higher than that of pure PLLA/PDLA homocrystals [90]. This scPLA forms a dense crystalline structure and a rigid amorphous fraction (RAF), effectively blocking gas diffusion pathways. These characteristics demonstrate the potential to overcome limitations of conventional PLA, such as oxygen and moisture barrier properties during high-temperature filling (hot-fill), microwave heating, and long-term distribution.

Early scPLA-based food packaging research focused on films using biofillers or natural separators such as cellulose and chitosan. The Katiyar group demonstrated that grafting a cellulose-based biofiller onto the PLA ROP process could simultaneously enhance the film’s mechanical strength and oxygen/moisture barrier properties by suppressing homocrystal formation and promoting SC crystal formation [164]. Additionally, research was reported using functionalized chitosan to induce SC crystal growth in PDLA/PLLA blends, simultaneously achieving reduced oxygen and water permeability along with enhanced antibacterial properties and hydrophilicity [165]. The Chen group systematically analyzed the mass transport properties and thermal characteristics of the scPLA structure in PDLA/PLLA blends. They demonstrated that the barrier properties are governed not only by crystallinity but also by the fraction of crystalline structures (α crystal, α′ crystal, and SC crystal) formed under various heat treatment conditions and the amount of RAF [133,166]. They emphasized that oxygen and moisture barrier properties vary significantly depending on the ratio of scPLA to α-crystallized hcPLA and that heat treatment history and cold crystallization conditions are key variables in packaging material performance design. Recently, SC films utilizing star-PDLA and linear PLLA blends have been reported [167], simultaneously enhancing SC crystal formation and processability, and high-heat-resistance/high-rigidity packaging films utilizing SC structures have been successively reported. While the scPLA structure remains a strategy for PLA-based food packaging materials, actual commercialization cases remain limited due to reasons such as reduced biodegradability from high crystallinity, the cost of high-purity PLLA/PDLA raw materials, process sensitivity, and film brittleness.

Meanwhile, research on PLA food packaging materials is gradually moving beyond improving the structure of a single polymer. It is evolving toward complementing PLA’s structural limitations through blending and copolymerization with other polymers possessing complementary properties [168]. Over the past decade, representative PLA blend partners have included thermoplastic starch (TPS), PHA (PHB/PHBV), PBAT, PBS, PCL, and natural polymers such as cellulose, chitosan, and proteins. Research has evolved toward designing combinations that balance stiffness, toughness, barrier properties, and functionalities (such as antimicrobial and antioxidant properties) [163,169].

The earliest focus of PLA blend research was systems utilizing starch and TPS. According to research by the Avérous group, PLA/TPS blends offer the advantages of low cost and excellent biodegradability. However, their sensitivity to moisture and mechanical limitations due to brittleness impose constraints on direct application as commercial packaging materials [170]. Subsequent studies aimed to overcome these limitations by designing a bilayer structure: instead of a single-layer blend, a TPS layer was laminated onto a PLA layer. The proposed structure suggests that the outer PLA layer manages moisture barrier and mechanical protection, while the inner TPS layer focuses on cost reduction and enhanced biodegradability [171].

Subsequently, research was conducted on blends with PHA (PHB/PHBV) to leverage PHB’s high crystallinity and barrier properties to compensate for PLA’s low barrier property and brittleness [117,172]. These studies assessed that PHB acts to inhibit PLA’s matrix, thereby mitigating PLA’s low moisture and oxygen barrier properties while simultaneously improving the elongation and impact strength of the PLA/PHB film. However, due to PHB’s low *T*_m_ (170–180 °C), PLA/PHB blends also exhibited a low *T*_m_ of 180–190 °C, resulting in consequent low strain at break [173]. Subsequently, introducing antimicrobial phenolic acids into PLA/PHBV blend films using PHBV, a PHA-based polymer, could impart antimicrobial functionality without significantly affecting biodegradability. This finding raised expectations for producing antimicrobial food packaging materials without compromising biodegradability [174].

PBAT is a representative partner polymer used to compensate for PLA brittleness. Despite being petroleum-based, PBAT is a biodegradable polymer exhibiting excellent ductility and tensile strength. Blending it with PLA can significantly enhance the elongation and toughness of the film. Studies show that small amounts of PBAT in PLA/PBAT blend films significantly enhance elongation at break with minimal strength reduction. Recently, active packaging systems incorporating antioxidant, antimicrobial, and UV-blocking functions have been actively researched by introducing various functional additives into PLA/PBAT blends [175,176,177]. Furthermore, PCL, a biodegradable polyester, exhibits a lower *T*_g_ and superior ductility compared to PBAT. PLA/PCL films are proposed for applications such as fruit and vegetable packaging, where toughness and durability in low-temperature environments are required [178].

Copolymer-based PLA packaging materials have recently garnered significant attention due to their ability to design *T*_g_, crystallinity, degradation rate, mechanical flexibility, and barrier properties through control of monomer composition [131]. For example, poly(LLA-co-caprolactone) (PLCL), which incorporates PCL as a copolymer, significantly enhances impact strength and elongation at break due to its low *T*_g_ and high ductility. By adjusting the CL content, oxygen and water vapor permeability, as well as interaction with moisture, can be precisely controlled. Therefore, it is expected that adjusting the CL ratio will enable broad application in the packaging field [137].

PLA–PEG block copolymers and composite films incorporating PEG enhance film flexibility and suitability for aqueous processes through PEG’s hydrophilicity and plasticizing effect. They can simultaneously impart oxygen and moisture barrier properties and functionalities (e.g., drug release, antimicrobial) by forming a hydrogel-like structure.

Copolymers with PGA are being investigated as alternatives to complement the barrier properties of PLA, leveraging PGA’s high barrier properties. The Gruter group reported that PLGA with high glycolic acid content can maintain melt processing similar to PLA while achieving oxygen and moisture barrier properties comparable to polyethylene terephthalate (PET) at room temperature [152]. They proposed that PLGA films, possessing both biodegradability and high barrier properties, could be a superior alternative to PLA films alone. However, excessive glycolic acid content in PLGA may accelerate glycolic acid hydrolysis in the presence of moisture, potentially compromising mechanical properties. Additionally, the high crystallinity in the copolymer form exhibits a tendency toward brittleness.

Finally, a strategy utilizing copolymer and blend structures of PBS and PLA has also been proposed. The Shibata group reported that blending Poly(butylene succinate-co-lactide) (PBSL)—a copolymer of biodegradable polyesters PLLA and PBS—with PLA allows simultaneous control of tensile strength, flexibility, and crystallization rate [179]. Chirachanchai et al. demonstrated for the first time that a random PBSL structure functions as a compatibilizer, plasticizer, and film clarity enhancer, improving the transparency and processability of PLA-based packaging films [180].

PLA-based food packaging materials have evolved from initial single PLA films to diverse design strategies, including SC structures, blends, copolymers, and multilayer film structures. Thus, structural design has become a key direction to enhance mechanical, thermal, and barrier properties while maintaining biodegradability and environmental friendliness. It also satisfies additional functionalities such as antimicrobial and antioxidant properties.

### 4.2. PLA Fiber

**Figure 8:**
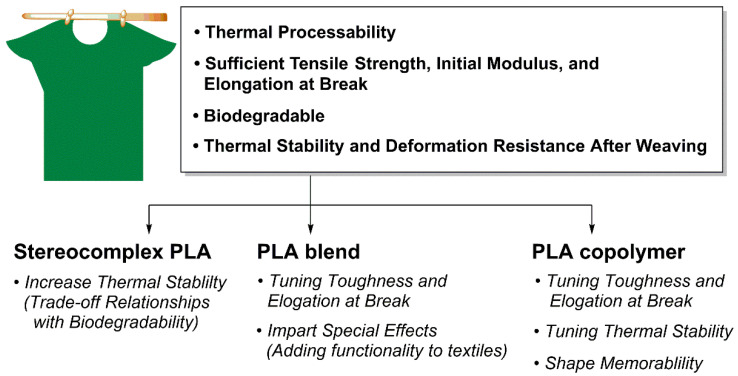
Required functional properties in fiber fields, and PLA-based strategies to achieve these requirements.

PLA is currently the only commercially available bio-based and biodegradable polymer that can be processed into fibers with sufficient mechanical strength via conventional melt-spinning techniques [181]. As a thermoplastic polyester, PLA can be melt-spun at elevated temperatures and, through appropriate drawing processes, can achieve the tensile strength and elasticity required for textile fibers (Figure 8). In addition, its low moisture absorption, low density, excellent UV resistance, and favorable optical properties associated with its low refractive index make PLA a promising bio-based alternative to conventional polyester fibers such as polyethylene terephthalate (PET) in textile and apparel applications [200].

Despite this potential, the broader adoption of PLA in fiber and textile applications is constrained by its relatively low thermal stability and narrow processing window. Thermal degradation during melt spinning or other high-temperature processing steps can lead to molecular weight reduction, which in turn adversely affects mechanical performance and long-term durability. Consequently, precise control of processing temperature, residence time, and moisture content is required during fiber production.

From an application perspective, PLA fibers must satisfy several key property requirements, including sufficient tensile strength, initial modulus, and elongation at break, to prevent filament breakage during spinning and drawing and to ensure adequate wear and fatigue resistance during use. Moreover, thermal dimensional stability above the glass transition temperature (*T*_g_) is essential to maintain shape stability during post-weaving heat-setting and high-temperature dyeing processes.

The early PLA fiber industry focused on biodegradable biomaterials. During the 1960s–1980s, numerous reports indicated that suture fibers such as VICRYL—a copolymer of glycolic acid and lactic acid (90:10 ratio)—could be applied as surgical materials due to their high biodegradability and biocompatibility [157,201]. By the 1990s–2000s, PLA for fibers and filaments became commercialized, establishing mass production processes for PLA fibers. This sparked serious discussions about expanding into the apparel and industrial fiber markets [202,203,204]. However, limitations such as PLA’s brittleness, low elongation, relatively poor impact resistance, and low heat resistance remain unresolved.

A representative strategy to overcome these limitations is blending and composite design, which involves adding other polymers or nanofillers during the melt-spinning process. PLA/PBS or PLA/PHBV blends are primarily used to enhance toughness and elongation. Various studies have reported that in PLA/PBS melt-spun filaments, increasing PBS content (0–12 wt%) leads to higher crystallinity, *T*_m_, and elongation at break, while also improving the fiber’s tensile strength and elastic modulus [205,206]. However, research by the Chen group indicated that while increasing the PHBV content in PLA/PHBV filaments within the range of 10–40 wt% improves fiber softness and tactile feel, excessive PHBV can reduce melt spinnability, mechanical strength, and elongation at break, making composition optimization necessary [207].

Furthermore, approaches have been reported to improve the eco-friendliness and tactile properties of PLA fibers by blending them with cotton or cellulose-based fibers or by adjusting surface properties through post-processing via wet processing [181]. In terms of imparting functionality, Doumbia et al. incorporated antibacterial properties using the antibacterial mechanism of ZnO in PLA/ZnO nanocomposite filaments, accepting a slight reduction in thermal and mechanical properties [208]. Annandarajah et al. also published research on manufacturing PLA fibers with mosquito repellent properties by blending the synthetic insect repellent N,N-diethyl-meta-toluamide during the extrusion process [209].

scPLA is gaining attention as a strategy to enhance the heat resistance of PLA-based fibers. A study on manufacturing scPLA fibers by melt-spinning a PLLA/PDLA blend reported that SC crystal formation raised the fiber’s *T*_m_ to approximately 220 °C and increased crystallinity, improving shape stability under high-temperature conditions such as heat setting and high-temperature washing [182]. The Marx group demonstrated that the crystallinity of melt-spun scPLA fibers increased from 50.5% to 59.7% compared to conventional semi-crystalline PLA fibers. Under identical spinning conditions, fiber-grade tensile strength of about 40–60 cN/tex was consistently achieved, indicating that scPLA may be suitable for high-strength technical fiber applications such as ropes [161].

PLA homopolymer fibers offer relatively high elastic modulus and tensile strength, but they are inherently brittle with low elongation. They also have limitations in dyeability due to their low heat distortion temperature, slow crystallization rate, and hydrophobic surface. The strategy to overcome these inherent limitations from a structural design perspective is to design PLA copolymer-based fibers. By controlling the types and sequence of copolymers, properties such as segment mobility, crystallization rate, hydrolysis sensitivity, *T*_g_, and *T*_m_ can be tailored to suit fiber applications. For example, ε-CL is a polymer with a very low *T*_g_ and high toughness. Block or random copolymerization with PLA can improve the flexibility and resistance to repeated deformation of the fiber. Pappalardo et al. compared PLLA-b-PCL and PDLLA-b-PCL block copolymers with PLLA-ran-PCL random copolymers, confirming that adding PCL segments not only improves rheological properties and mechanical flexibility but also gives the material shape-memory properties [183]. In a study of medical sutures using an LLA: ε-CL = 70:30 random copolymer (PLC), primary hot-drawing reduced stiffness and increased flexibility, while secondary hot-drawing restored tensile strength through the formation of a semicrystalline structure. Additionally, drug coating could be applied to impart wound-healing promotion functionality [184].

Copolymers are also utilized to adjust PLA’s relatively low heat deflection temperature and slow crystallization rate up or down to suit specific applications. In particular, random copolymers are a useful strategy for controlling thermal properties because the introduction of random monomers within the polymer chain disrupts chain regularity, reducing PLA crystallinity and lowering its melting point. For example, GA, being smaller and more polar than LA, reduces crystallinity when forming a random copolymer with PLA and simultaneously modulates *T*_g_, *T*_m_, thermal stability, and degradation rate. This is evident from the single, double, or triple melting peaks observed in DSC measurements, depending on the copolymer composition and crystallization conditions [185]. As glycolide (GA) content increases, *T*_g_ and *T*_m_ decrease, crystallinity reduces, and hydrolysis rate tends to increase. The lower *T*_m_ enables melt-spinning at lower temperatures, offering the advantage of reducing thermal decomposition during processing [186]. Furthermore, TMC is an aliphatic carbonate with amorphous properties and a very low *T*_g_. Similar to GA, it strongly reduces *T*_g_, *T*_m_, and crystallinity of PLA through random copolymer formation, enabling the creation of very soft thermoplastic materials. In their study on poly(LLA-co-trimethylene carbonate) fibers, Fuoco et al. systematically analyzed the evolution of the crystalline structure from the α′-form to the α-form, changes in crystallinity, and tensile properties based on copolymer composition, melt-spinning, and hot-drawing conditions. They presented a composition–process–structure–property correlation enabling the design of fiber mechanical properties and thermal stability [187].

In the PLA-based fiber and fabric field, scPLA, blends, and copolymer design are all used, with each strategy selectively applied based on target properties such as heat resistance, toughness, elongation, and functionality. Future research must combine copolymer design with multiscale process control to establish a PLA-based fiber platform with durability and functionality competitive with existing PET and PE fibers, remaining a critical challenge.

### 4.3. Agriculture

In agricultural applications such as mulch films, greenhouse coverings, and controlled-release systems, material selection must balance durability during use with controlled degradation after service life, while also considering cost competitiveness. In this context, polylactic acid (PLA), a bio-based polyester derived from renewable resources such as corn and sugarcane, has attracted attention as a candidate material for agricultural products owing to its biodegradability, relatively good gas-barrier properties, light transmittance, and moderate stiffness and modulus [210]. In particular, the transparency, low moisture uptake, and biodegradability of PLA under soil or composting conditions make it a meaningful circular alternative to conventional polyethylene (PE) films, which typically require collection and incineration after use.

Despite these advantages, the practical application of PLA in agricultural films has been limited by its relatively low heat distortion temperature, slow crystallization rate, and intrinsic brittleness. These characteristics make it challenging to achieve sufficient tear strength, elongation, and fatigue resistance to withstand mechanical stress throughout an entire growing season without film rupture. Moreover, the higher material cost of PLA compared to polyethylene or polypropylene further restricts its direct substitution in cost-sensitive agricultural applications.

**Figure 9:**
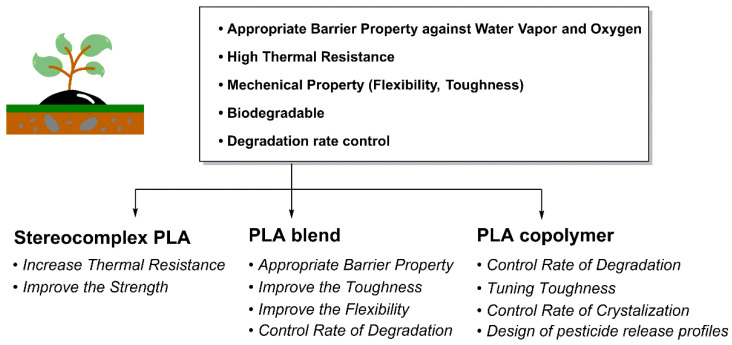
Required functional properties in agriculture, and PLA-based strategies to achieve these requirements.

Accordingly, the technological development of PLA for agricultural use can be broadly divided into three stages: early trials employing neat PLA films with limited performance, subsequent implementation of blend-based formulations to improve toughness and processability, and, more recently, the development of high-performance materials based on stereocomplex formation and copolymer design that enable more precise control of mechanical properties and degradation behavior (Figure 9) [148]. These advances reflect ongoing efforts to optimize PLA-based systems for agricultural applications rather than pursuing simple one-to-one replacement of conventional polyolefin films.

The most direct strategy to complement PLA’s weaknesses has been blending with flexible biodegradable polyesters. Representative examples include blends of PLA with PBAT [188], PBS [189], and PCL [113,115], which have become established as formulations to significantly improve toughness and elongation while largely retaining the stiffness and barrier performance of PLA. Numerous studies on PLA/PBAT, PLA/PBS, and PLA/PHA blends have quantitatively shown that increasing the PBAT or PBS content enhances tensile elongation at break and tear strength and improves film processability, but that excessive amounts of the soft phase lead to reductions in stiffness and heat distortion temperature [190]. From the standpoint of agricultural mulching films, the key challenge has been to exploit this trade-off to identify an optimal composition that is robust enough during the cultivation period but fractures and degrades rapidly after harvest. According to Yang et al., in field studies applying PLA/PBAT-based biodegradable mulching films to winter potato cultivation in southern China, soil temperature, moisture retention, and early growth promotion were comparable to or better than those obtained with PE films [191]. Over the course of two cropping seasons (winter–spring), the films gradually degraded so that, when incorporated into the soil during post-harvest plowing, they did not exert any negative effects on the growth or yield of subsequent crops. Later work introduced glycidyl methacrylate-based chain extenders and reactive compatibilizers to control phase-separated morphology and interfacial structure within the blend [189] and added fillers such as cellulose nanofibers, plant residues, and starch to simultaneously improve elongation, strain at break, and moisture retention while maintaining tensile and tear properties [192]. Athanassiou et al. showed that PLA-based mulching films containing various types of non-food plant by-products as fillers can maintain high extensibility, stretch uniformly over the soil surface to provide the flexibility required for crop cultivation, and, after use, be safely incorporated into the soil together with the fillers [193]. Thus, PLA blends represent the practical technological axis that has elevated PLA from a laboratory-scale biodegradable film to a material that can be deployed as a functional agricultural film in real fields.

Whereas blends have primarily addressed mechanical limitations such as toughness and elongation, SC formation has emerged as a strategy to fundamentally enhance the thermal robustness of PLA. scPLA consists of regularly interpenetrating PLLA and PDLA chains and forms crystals with melting temperatures approximately 50 °C higher than those of PLLA or PDLA homocrystals, reaching around 210–230 °C [89]. The associated increase in crystallinity leads to improved thermal stability and mechanical strength at elevated temperatures [12]. These features are particularly attractive for agricultural materials that must withstand prolonged exposure to hot soils during summer or long-term solar irradiation in applications such as greenhouse coverings and tunnel films. For example, Gupta et al. prepared scPLA nanobiocomposites by melt-extruding PLLA/PDLA blends with nano-sized hydrophobic/hydrophilic micellar chitosan (MCH) to further enhance the high-temperature stability of scPLA. MCH increased particle surface area and converted chitosan into nanosized amphiphilic domains that efficiently promote SC crystal formation [194]. The resulting scPLA/MCH composites exhibited a marked increase in heat deflection temperature from about 70 °C for neat scPLA to above 140 °C, accompanied by simultaneous increases in tensile strength and modulus, thereby showing a clear improvement in overall thermo-mechanical stability. Although such high-heat-resistance scPLA formulations can be used as stand-alone films, in practical agricultural applications, they are often employed in blends with PBAT or PBS, where SC domains form within the blend matrix. In these systems, films are designed to maintain high stiffness and heat resistance early in use and then gradually degrade under the combined influence of hydrolysis and microbial activity toward the end of the growing season and after harvest [195]. In other words, scPLA functions as a high-heat, high-strength component that enhances the heat resistance of PLA mulching films. When combined with the softer phases of the blend, it serves as a crucial structural element for designing durable agricultural materials that perform well under high temperatures and solar irradiance until harvest.

Finally, PLA copolymers have evolved beyond simple tuning of strength and heat resistance to become tools for “designing” the service life, degradation rate, and functionality of agricultural materials. In previous studies, partner monomers such as glycolide [160], ε-CL [196], and TMC [197] were incorporated to adjust PLA’s *T*_g_, *T*_m_, crystallinity, and hydrophilicity so that mulching films and pot materials would remain intact only for a period matched to soil temperature, moisture, and crop growth duration. Among these, PLGA, obtained by copolymerizing GA with lactic acid, is well known for enabling precise control of in vivo degradation time from several weeks to several months by tailoring comonomer composition [160]; this concept of tuning degradation behavior and thermal properties has been proposed as an applicable strategy for mulching films and pot materials that must retain their properties only over the cultivation period. Incorporating relatively flexible partner monomers such as ε-CL or carbonate units lowers *T*_g_ and modulus, allowing films and coatings to better withstand repeated deformation, while block or multiblock architectures can be used to generate microphase separation between rigid PLA segments and soft segments, thereby balancing mechanical strength and flexibility. In parallel, there have been efforts to exploit PLA copolymers as carriers for agrochemical delivery [196,197]. PLGA, composed of lactide and glycolide, has been utilized as a nanocarrier platform in “nano-pesticide” delivery systems, encapsulating active ingredients such as herbicides and fungicides in nano- or microparticles to protect them, facilitate transport, and improve mobility and controlled release in biological systems, drawing on its tunable degradation rate and drug-release profile [198].

In summary, agricultural applications of PLA have evolved from relying solely on the intrinsic biodegradability and barrier properties of neat PLA films to securing the mechanical stability and processability required for field use through blend technologies, and further to finely controlling heat resistance, degradation rate, and functional performance via SC structures and copolymer design. In the future, it is probable that structural and processing strategies will be more specifically designed for particular crops, regional climate conditions, and variations in soil microbial communities, resulting in the creation of PLA-based agricultural materials that are tailored to specific crops and regions.

## 5. Summary and Outlook

PLA is a representative bio-based thermoplastic polymer derived from lactic acid, produced from renewable resources such as corn and starch. It is a polymer that satisfies three key characteristics: biocompatibility, biodegradability, and bio-based origin. Additionally, it possesses relatively superior mechanical strength, transparency, and thermoplasticity. This makes it a strong candidate to replace petroleum-based plastics in applications such as food packaging, textiles, agricultural mulching films, medical devices, and drug delivery materials. Recently, demand for biodegradable plastics has been rapidly increasing due to stricter regulations on single-use plastics and carbon neutrality policies. PLA is currently the most prominent biodegradable polymer, suitable for large-scale production and diverse applications.

This paper systematically reviews PLA-based biodegradable polymers “from synthesis to application” within this context. First, it summarizes the manufacturing processes for lactic acid and lactide monomers, along with the PLA synthesis mechanisms based on direct condensation polymerization and ROP, and the effects of catalysts or reaction conditions on molecular weight and stereoregularity. Recent studies have aimed to improve PLA’s heat resistance, mechanical properties, and biodegradability while adding functionalities through structural design, including scPLA, PLA copolymers, and blends. This reveals diverse strategies to overcome the limitations of conventional PLA. Finally, by focusing on the properties required for PLA in its practical application fields, various application cases such as packaging, textiles and apparel, agriculture, and drug delivery systems are summarized, presenting the linkages between structure, process, property, and application.

Nevertheless, numerous challenges remain to be addressed before PLA can establish itself as a true universal substitute for petroleum-based plastics. Limitations such as its still-low *T*_g_, restricted heat resistance, brittleness, and insufficient moisture and oxygen barrier properties constrain its expansion into high-value packaging, textiles, and industrial materials. Furthermore, the understanding of its degradation behavior in real environments, such as soil or oceans, and the potential for microplastic formation remains incomplete. Additionally, developing commercialization strategies for chemical and physical recycling processes and blend systems remains an ongoing challenge. Future PLA research should proceed in the following directions: (i) developing eco-friendly catalysts and processes that minimize metal residues during polymerization, (ii) achieving materials with high heat resistance and moisture resistance, and (iii) designing environmentally triggered degradation systems and recycling systems. Concurrently, securing long-term durability in new application areas such as electronic devices, 3D printing, and construction must be pursued. As research advances with the help of industrial infrastructure and policy support, PLA can position itself not just as an alternative to petroleum-based plastics but as a fundamental component of a sustainable plastic circular economy.

## Figures and Tables

**Table 1:** Comparison of PLA synthesis routes and catalyst systems.

Catalyst System	Typical Conditions	Achievable Mw (kg/mol)	Polydispersity (Đ)	Stereocontrol	Toxicity	Scalability & Maturity	References
Sn(Oct)_2_	130–180 °C	100–320	Broad	Limited	Residual Sn (medical restriction)	Industrial benchmark	[78,79,80,81,82]
Zn-based complexes	20–160 °C	50–130	1.05–1.70	Good	Low toxicity	Lab-pilot	[29,30,31,32,33]
Al-based complexes	70–120 °C	10–60	1.10–1.30	Excellent	Low toxicity	Lab–scale	[37,38,39,83,84,85]
Organocatalysts (DBU, TBD, Thiourea)	−25 °C	10–100	1.05–1.25	Moderate	Metal-free	Lab-scale	[43,44,45,47,48,49,51,52,53,54,55]
Enzyme catalysts (CAL-B, BCL, PPL)	50–130 °C	<70	Broad	Moderate	Metal-free/Green	Limited (low yield, long reaction time)	[63,66,67,69]
Lewis pair/FLP	40–110 °C	30–40	1.10–1.40	Limited	Low toxicity	Lab-scale (emerging)	[71,72,75,76,77]
Direct polycondensation	130–180 °C	20–50	Broad	Poor-Moderate	Metal catalyst residue	Limited	[12,13,14,15,16,86,87]

**Table 2:** Modification strategies of PLA and property trade-offs.

Modification Strategy	Toughness	Heat Resistance	Barrier Properties	Biodegradability	Processability	Key Trade-Offs	References
Neat PLA	Low	Low	Moderate	Moderate	Moderate	Brittleness	[94,130,131,132]
Stereocomplex PLA (scPLA)	Low–moderate	Very high	High	Low	Poor	Processing	[90,91,92,96,97,98,133]
PLA/PCL blends	High	Low	Low	High	Good	Strength	[110,113,115]
PLA/PHB blends	Low	Moderate	High	High	Moderate	Brittleness	[105,116,117]
PLA/PBAT blends	Very high	Low	Moderate	High	Good	Strength	[125,126,134]
PLA/PCL copolymers	High	Tunable	Low-Moderate	High	Good	Strength	[106,135,136,137,138]
PLA/PEG copolymers	High	Low	Moderate	High	Good	Moisture sensitivity	[139,140,141,142]
PLA/PGA copolymers	Low	High	Very high	Tunable	Poor	Brittleness	[143,144,145,146]

**Table 3:** Biodegradation behavior of PLA-based systems under different environments.

Material System	Environment	Conditions	Degradation Metric	Time Scale	References
Neat PLA	Compost	58 °C	34.4% disintegration (% mass loss, DIN EN ISO 20200)	3 months	[161]
Neat PLA	Soil	22 ± 2 °C	No *M*_n_ change (≤3 months) *M*_n_ ≈ 50% (24 months)	24 months	[162]
Neat PLA	Marine	25 °C	No major macroscopic change (≤4 months) slow *M*_n_ decrease thereafter	10 months	[130]
scPLA	Compost	58 °C	28.6% disintegration (% mass loss, DIN EN ISO 20200)	3 months	[161]
PLA/PCL	Marine	30 °C	11% *M*_n_ remaining (ASTM D6691–based seawater)	6 months	[136]
PLA/PEG	Marine	20 °C	72.63% biodegradation, 71.5% mass loss (BOD, OECD 306)	28 days	[140]
PLA/PGA	Marine	25 °C	*M*_n_ decreases faster than neat PLA (rate increases with GA content)	4–10 months	[130]

**Table 4:** Application-driven selection guide for PLA materials.

Application	Key Requirements	PLA Design	References
Food packaging	Gas and Water barrier properties, Thermal Resistance, Toughness, Biodegradable	ScPLA, Blend (TPS, PHB, PBAT) Copolymer (PCL, PEG, PGA, PBS)	[133,137,164,165,166,167,168,169,170,171,172,173,174,175,176,177,178,179,180]
Fiber and textile	Processability, Toughness, Biodegradable, Thermal Stability	ScPLA, Blend (cotton, cellulose-based fibers) Copolymer (PCL, PGA)	[161,181,182,183,184,185,186,187]
Agriculture	Thermal Resistance, Toughness, Controlled degradation	ScPLA, Blend (PBAT, PBS, PCL, PHA, PBS) Copolymer (PCL, PGA, succinate, trimethylene carbonate)	[12,89,113,115,188,189,190,191,192,193,194,195,196,197,198]

## Data Availability

No new data were created or analyzed in this study.
